# Fine-tuning striated muscle performance: conserved sarcomere-level mechanisms across insect and vertebrate systems

**DOI:** 10.3389/fphys.2026.1836341

**Published:** 2026-06-18

**Authors:** Hiroyoshi Matsui, Zach Chbihi, Rima Mashni, Haidar Hachem, Sarah Snider, Alyson Sujkowski

**Affiliations:** 1 Wayne State School of Medicine Department of Pharmacology, Detroit, MI, United States; 2 University of Maryland Baltimore County College of Natural and Mathematical Sciences, Baltimore, MD, United States; 3 Wayne State School of Medicine Eugene Applebaum College of Pharmacy and Health Sciences, Detroit, MI, United States

**Keywords:** Drosophila models, Frank-Starling mechanism, indirect flight muscle, muscle disease, myofilament regulation, sarcomere elasticity, stretch activation, titin-like proteins

## Abstract

Striated muscles exhibit remarkable structural and functional specialization that enables precise control of force production, contractile kinetics, and energetic efficiency. Although vertebrate skeletal and cardiac muscles have been extensively studied, comparative analyses across animal phyla reveal that many molecular and biophysical principles governing muscle performance are deeply conserved. Insects, in particular, possess highly differentiated muscle types that provide powerful systems for dissecting the regulation of contraction, elasticity, and force generation at the level of the sarcomere. In this review, we integrate insights from insect and vertebrate muscles to highlight conserved and divergent features of sarcomere organization and myofilament composition. We focus on major contractile and regulatory proteins, including actin, myosin, troponin, tropomyosin, and elastic proteins, emphasizing how isoform diversity fine-tunes muscle function. We discuss the biomedical relevance of invertebrate models for understanding muscle disease mechanisms, including congenital myopathies, sarcomeric protein-associated disorders, and muscular dystrophies. Finally, we examine how principles uncovered in insect muscles inform vertebrate cardiac physiology and skeletal muscle aging, positioning insect systems as complementary discovery platforms for advancing muscle biology.

## 1 Introduction

Striated muscles in vertebrates include cardiac and skeletal muscles and are defined by the organization of contractile proteins into sarcomeres, producing the characteristic striped appearance observed under the microscope (Chemello et al., 2023). The contractile force generated by striated muscle is crucial for powering mobility, postural control, respiration, and circulation (Shadrin et al., 2016). Achieving this force while meeting the substantial energetic demands of contraction requires precisely regulated molecular mechanisms within the sarcomere. The importance of these regulatory pathways is underscored by their high degree of evolutionary conservation from invertebrates to humans (Cao et al., 2019; Cao et al., 2020). Indeed, many fundamental principles of muscle performance were first uncovered in flying insects. Studies using invertebrate models such as *Drosophila* have provided key insights into the physiological and genetic mechanisms that govern myofilament homeostasis and muscle aging, as well as disease-associated changes in skeletal and cardiac muscle (Blice-Baum et al., 2019). In this review, we examine how studies of insect myofilament structure and force production reveal conserved sarcomere-level mechanisms that shape muscle performance across species and inform our understanding of vertebrate muscle physiology and disease.

## 2 Highly differentiated structure and function of striated muscles conserved in vertebrates and invertebrates

Muscle power, efficiency, structure, and Ca^2+^ dynamics are shaped by tissue-specific differences in myofilament isoform expression and stoichiometry. In vertebrates, this diversity is exemplified by the presence of slow-twitch (type I) and fast-twitch (type II) skeletal muscle fibers, which differ in metabolic properties and contractile kinetics (Schiaffino and Reggiani, 2011). Slow-twitch fibers rely primarily on aerobic metabolism to support sustained activity, whereas fast-twitch fibers utilize anaerobic glycolysis to generate short, high-power contractions (Schiaffino and Reggiani, 2011). Cardiac muscle further exemplifies specialization, expressing distinct contractile protein isoforms that support continuous, involuntary contraction throughout life. Insect muscles display comparable functional diversity, with pronounced differentiation in myofilament composition to meet distinct physiological demands (Laurichesse and Soler, 2020).

The major myofiber types in *Drosophila* include cardiomyocytes, fibrillar muscles, and tubular muscles, each with distinct structural and functional properties (Ocorr et al., 2014; Laurichesse and Soler, 2020). In *Drosophila*, the adult heart consists of a dorsally positioned linear cardiac tube composed of approximately 80 myogenically derived cardiomyocytes (Rotstein and Paululat, 2016). Contractility is coordinated by two myogenic pacemakers, with the dominant pacemaker determining the direction of hemolymph flow (Dulcis and Levine, 2005; Wasserthal, 2007). Despite its structural simplicity, the *Drosophila* heart exhibits strong conservation with vertebrate cardiac muscle at the levels of gene sequence, epigenetic regulation, proteostasis, Ca^2+^ cycling, metabolism, and extracellular matrix organization (Brown and Cohen, 2005).

Adult somatic muscles in *Drosophila* are broadly classified into tubular and fibrillar types based on morphology and contractile properties (Laurichesse and Soler, 2020). Tubular muscles, characterized by fibers arranged around a central lumen, include abdominal muscles (Middleton et al., 2006; Chakravorty et al., 2012), leg muscles (Soler et al., 2004), jump muscles (Trimarchi and Schneiderman, 1995), and direct flight muscles (Trimarchi and Schneiderman, 1995). Fibrillar muscles comprise the thoracic indirect flight muscles (IFM), characterized by unbundled myofibrils that remain discrete and distributed throughout the myoplasm without fusing into traditional bundles. In addition, fibrillar IFM have exceptionally high mitochondrial content and reduced sarcoplasmic reticulum (Josephson et al., 2000). These features give rise to specialized myofibrils with distinct ultrastructure and bioenergetic properties.

Fibrillar IFM from *Drosophila* and other insects, including *Apis*, *Anopheles*, and *Lethocerus*, have proven especially useful for investigating myofilament regulation and mechanically driven force production. Comparative studies across these systems highlight both the conservation of core sarcomeric components and the specialization of regulatory and elastic elements that support distinct contractile behaviors.

## 3 Synchronous and asynchronous contractions

The fundamental contractile unit of striated muscle in both vertebrates and invertebrates is the sarcomere, composed of interdigitating myosin thick filaments and actin thin filaments (Paniagua et al., 1996). Force is generated through cyclic interactions between myosin heads and actin, powered by myosin ATPase activity. This process is regulated by Ca^2+^ through the thin-filament associated troponin-tropomyosin (Tn-Tm) complex (Bullard and Pastore, 2011; Sheng and Jin, 2014).

**Figure 1:**
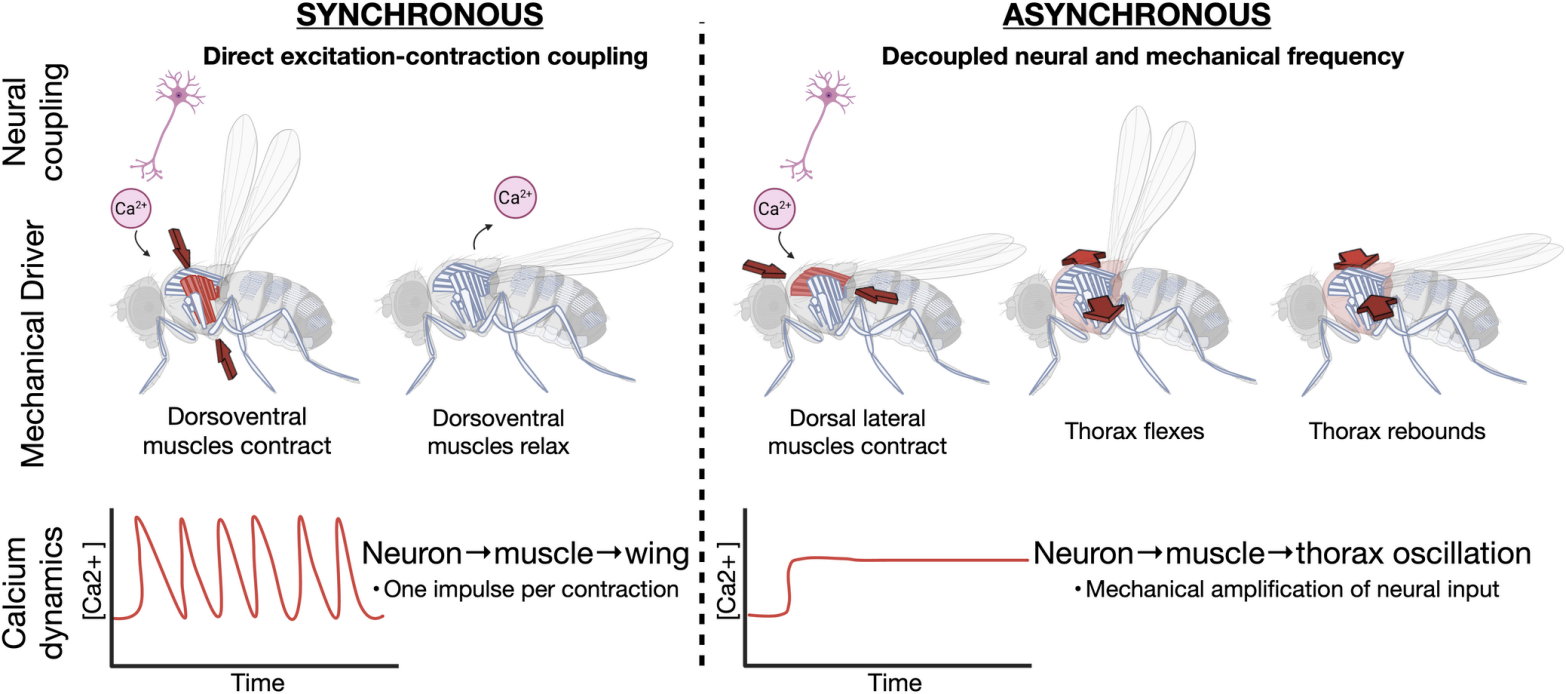
Mechanical and calcium signaling differences between synchronous and asynchronous insect flight muscle. Schematic comparison of synchronous (left panel) and asynchronous (right panel) insect flight muscle highlighting differences in neural coupling, mechanical activation, and calcium dynamics. In synchronous muscle, each motor neuron impulse generates a discrete Ca^2+^ transient that activates the troponin-tropomyosin complex, producing a single contraction-relaxation cycle and a 1:1 relationship between neural input and mechanical output. Contraction is directly coupled to Ca^2+^ cycling. In contrast, asynchronous indirect flight muscle (IFM) operates at sustained, permissive Ca^2+^ levels that maintain partial activation of the contractile apparatus. Low-frequency neural input initiates oscillatory contractions maintained through stretch activation, in which thoracic deformation mechanically activates antagonistic muscles. Phasic Ca^2+^ spikes in synchronous muscle contrast with tonic priming Ca^2+^ levels in asynchronous IFM, enabling high-frequency, energy-efficient contraction. Created in BioRender. Sujkowski, A. (2026) https://BioRender.com/ntbo1ok.

In resting muscle, cytoplasmic Ca^2+^ levels are low, and the Tn-Tm complex sterically blocks myosin-binding sites on actin (Brown and Cohen, 2005). Following myocyte depolarization, Ca^2+^ is released into the cytoplasm and binds troponin, inducing a conformational change that shifts tropomyosin away from these binding sites (Mckillop and Geeves, 1993; Lehman and Craig, 2008). Myosin cross-bridge formation further stabilizes the open state of the thin filament, enabling full activation and contraction (Vibert et al., 1997; Cammarato et al., 2010). In synchronous muscles, each contraction-relaxation cycle is tightly coupled to a single action potential (**Figure 1**).

Insects possess an additional mode of contraction that partially decouples mechanical output from direct neural control. In asynchronous muscle, force generation does not occur in a 1:1 relationship with action potentials. Flight imposes extreme energetic demands, and the kinetics of Ca^2+^ release and reuptake are insufficient to support the very high oscillatory frequencies required for wing movement. In addition, continuous sarcoplasmic reticulum-dependent Ca^2+^ cycling at these frequencies would be metabolically costly (Josephson et al., 2000). Instead, asynchronous IFM rely on stretch activation (SA), a process in which mechanical deformation of activated muscle promotes delayed force generation. As a result, multiple contraction cycles can occur following a limited number of neural impulses (Pringle, 1978). In flies, IFM are organized into two opposing muscle groups, the dorsal longitudinal muscles (DLMs) and dorsal ventral muscles (DVMs), whose alternating contractions deform the thoracic exoskeleton (**Figure 1**). During flight, alternating activation of antagonistic muscle groups deforms the thorax, producing self-sustaining mechanical oscillations that drive rapid wingbeats.

Stretch activation operates within a permissive range of myoplasmic Ca^2+^ concentrations that maintains partial activation of the contractile apparatus (Linari et al., 2004; Bullard and Pastore, 2011; Bullard and Pastore, 2019). Within the physiological range examined experimentally (approximately pCa 5.7–5.4), power output generally increases with increasing Ca^2+^ concentration (Wang et al., 2011; Glasheen et al., 2017). Maximal power output in *Drosophila* and *Lethocerus* IFM occurs near saturating Ca^2+^ levels (approximately pCa ~5.0) (Glasheen et al., 2017), although some studies suggest that at very high activation levels, increased isometric tension may reduce the relative contribution of stretch activation to net power generation (Wang et al., 2011). Importantly, asynchronous flight muscle does not uniquely enable high-frequency contraction, as synchronous flight muscles in other insects can also operate at relatively high frequencies. Rather, the defining feature of asynchronous IFM is the decoupling of neural firing frequency from mechanical oscillation frequency. Motor neurons fire at comparatively low frequencies (e.g., ~5 Hz) to maintain a permissive Ca^2+^ state, while the thoracic-muscle system oscillates mechanically at much higher frequencies (e.g., ~200 Hz) (Wang et al., 2011; Glasheen et al., 2017). Modulation of myoplasmic Ca^2+^ levels further tunes force production and contributes to adjustments in wing stroke amplitude and frequency under different flight conditions (Boettiger and Furshpan, 1952; Gordon and Dickinson, 2006).

Stretch-activated muscles exhibit several distinct forms of mechanically modulated force production that differ in their physiological roles, timing, and proposed molecular basis. In insect flight muscle, SA refers to the delayed increase in force that follows rapid muscle lengthening, whereas shortening deactivation (SD) describes the delayed reduction in force following rapid muscle shortening (Loya et al., 2022). Together, these complementary processes coordinate oscillatory contraction by enhancing positive work during shortening while minimizing resistive forces during subsequent lengthening (Josephson, 1999; Josephson et al., 2000; Loya et al., 2022). Recent evidence further suggests that muscle-specific myosin isoforms can modulate these responses, as expression of the *Drosophila* cardiac myosin isoform CardM2 increases the magnitude of both SA and SD tension in jump muscle (Bell et al., 2025).

Tight temporal coordination between SA and SD is therefore essential for maximizing net mechanical power output during high-frequency wingbeat cycles (Steiger, 1977; Josephson et al., 2000). In both *Drosophila* and *Lethocerus* IFM, SA and SD exhibit similar calcium dependence but differ in amplitude and timing (Hisey et al., 2009). Compared with SA, SD occurs earlier in the contractile cycle, with greater temporal separation observed in *Lethocerus*, possibly reflecting adaptation to its lower wingbeat frequency and longer contraction cycle (Loya et al., 2022). Although SA has been extensively studied for decades, SD remains comparatively less characterized experimentally.

In addition to oscillatory phenomena such as SA and SD, muscles can also exhibit history-dependent increases in force following active lengthening, termed residual force enhancement (rFE). Unlike SA and SD, which contribute to cyclical oscillatory contraction, rFE reflects a sustained increase in force following active lengthening. rFE is defined as the elevated steady-state force observed after active stretch compared with the purely isometric force produced at the same final muscle length (Herzog, 2014). Current evidence suggests that rFE arises from emergent sarcomeric properties involving interactions among titin (Fukuda and Granzier, 2005), cross-bridges (Campbell et al., 2018), and filament lattice structure (de Tombe et al., 2010), although the relative contributions of these components remain under investigation (Hisey et al., 2009; Joumaa and Herzog, 2013; Herzog, 2014). The existence of rFE highlights limitations of classical cross-bridge theory, which does not fully account for the sustained increase in force observed following active muscle lengthening. Although the molecular basis of rFE remains unresolved, IFM may provide a useful model system for investigating the structural determinants of history-dependent force production.

Length-dependent modulation of force production is also a defining feature of vertebrate cardiac muscle (Stelzer and Moss, 2006; Shiels and White, 2008; Kawai and Jin, 2021). In the heart, increased preload enhances force generation through the Frank-Starling mechanism (Delicce and Makaryus, 2021), a phenomenon that allows cardiac output to adjust dynamically to ventricular filling. Although SA, SD, rFE, and Frank-Starling behavior differ in timescale, physiological context, and mechanical output, these phenomena collectively illustrate the importance of mechanically regulated force production in muscle contraction.

Multiple, non-mutually exclusive models have been proposed to explain stretch activation in IFM. These include strain-dependent modulation of cross-bridge kinetics (Granzier and Wang, 1993), increased proximity of actin target sites to myosin heads during stretch (Wray, 1979), and mechanical coupling between thick and thin filaments through regulatory complexes or myosin light chain interactions (Reedy et al., 1994; Tohtong et al., 1995). Recent structural and computational studies further support a more integrated framework in which both thin- and thick-filament mechanisms contribute. Current models of stretch activation in IFM propose coordinated contributions from both thick- and thin-filament regulatory systems. On the thick-filament side, force-dependent recruitment of myosin heads from the inactive super-relaxed state (SRX) may provide a strain-dependent link between sarcomere strain and contractile activation (Campbell et al., 2018). Structural studies further suggest that stretch-dependent transitions within the interacting-head motif (IHM) regulate the availability of actin-binding myosin heads during oscillatory contraction (Hu et al., 2017; Bullard and Pastore, 2019). These observations are consistent with models in which strain-induced changes in thick-filament structure contribute to strain-dependent modulation of force production during oscillatory contraction.

On the thin-filament side, time-resolved structural and X-ray diffraction studies indicate that stretch is associated with tropomyosin movement, cross-bridge binding, and force development in IFM (Perz-Edwards et al., 2011; Bullard and Pastore, 2019). These findings are consistent with steric blocking–unblocking models in which tropomyosin regulates access to actin-binding sites and suggest the presence of strain-sensitive regulatory structures at the level of troponin.

Some studies have further proposed the existence of persistent cross-bridges outside the target zone, termed troponin bridges, which may contribute to tropomyosin displacement during stretch (Wu et al., 2010; Perz-Edwards et al., 2011; Bullard and Pastore, 2019). In this model, Ca^2+^ serves a permissive role by relieving inhibitory constraints on the thin filament, whereas mechanical stretch promotes tropomyosin movement and cross-bridge recruitment. However, the mechanistic contribution of troponin bridges remains under investigation, and multiple competing models for SA likely coexist.

**Figure 2:**
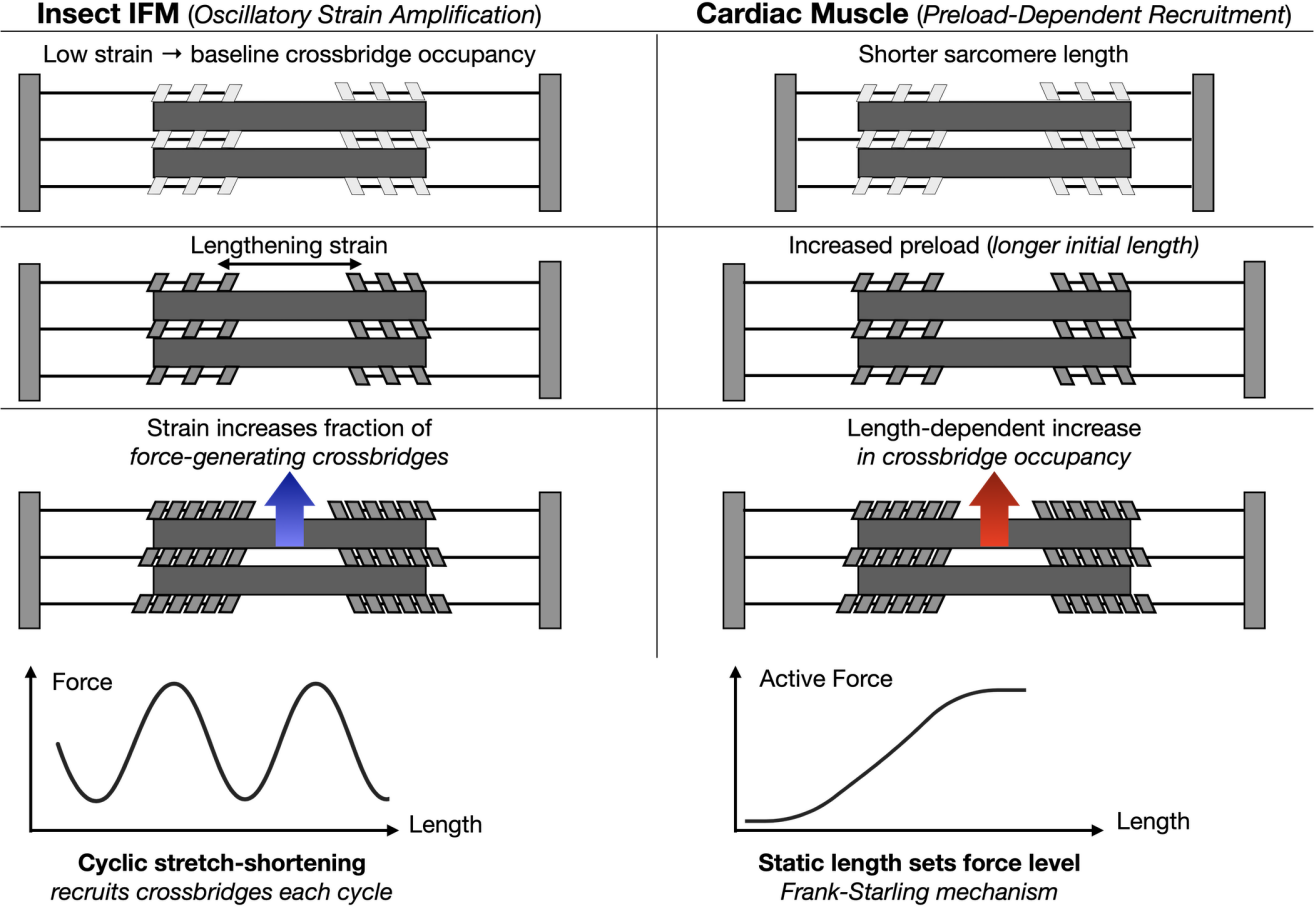
A conserved sarcomere-level mechanism underlies oscillatory power in IFM and length-dependent activation in cardiac muscle. Lengthening strain increases the fraction of force-generating cross-bridges within evolutionarily conserved sarcomeric architectures (depicted as increased density of attached heads). Z-disc separation indicates increased sarcomere length. In insect indirect flight muscle (IFM), cyclic stretch-shortening modulates cross-bridge recruitment during each oscillation, amplifying power output. In cardiac muscle, increased diastolic length (preload) enhances systolic force through length-dependent activation (Frank-Starling mechanism). The underlying strain-sensitive principle is shared, but its physiological deployment differs.

Additional structural changes, including lattice compression and thick-filament twisting, accompany stretch and are thought to modulate rather than directly control force generation (Campbell et al., 2018). Importantly, simulations suggest that force-dependent myosin recruitment and thin-filament cooperativity act synergistically to produce length-dependent force, as disruption of either mechanism impairs force generation or slows relaxation at extended sarcomere lengths (Campbell et al., 2018). Although differing in mechanistic detail, these models converge on the idea that coordinated interactions between thin- and thick-filament systems regulate oscillatory force generation (**Figure 2**).

Regulation of contraction through coordinated thin- and thick-filament interactions appears to operate through conserved molecular principles in both insect and vertebrate muscle (Lehman and Craig, 2008). Although IFM proteins possess specialized adaptations that support rapid oscillatory contraction, genetic, structural, and biophysical studies have revealed substantial conservation in protein-protein interactions, regulatory kinetics, and load-dependent behavior across stretch-activated muscle systems.

In vertebrate cardiac muscle, force production increases with sarcomere length through the Frank-Starling mechanism (Fukuda and Granzier, 2005; de Tombe et al., 2010; Ma et al., 2021). Current models suggest that this response involves coordinated contributions from enhanced thin-filament activation, strain-dependent recruitment of myosin heads, and modulation of elastic protein stiffness (Fukuda and Granzier, 2005; de Tombe et al., 2010; Ma et al., 2021). Mechanical stretch can also directly modulate cardiac force production under certain conditions, suggesting the presence of intrinsic stretch-sensitive regulatory mechanisms beyond simple filament overlap (Vemuri et al., 1999; Stelzer and Moss, 2006). Physiological factors including preload, afterload, inotropic state, and autonomic signaling alter the Frank-Starling relationship, whereas pathological conditions such as systolic heart failure and dilated cardiomyopathy impair its effectiveness (Delicce and Makaryus, 2021). Although compensatory length-dependent activation can partially preserve cardiac output, failure of this adaptive mechanism contributes significantly to cardiac dysfunction (Delicce and Makaryus, 2021).

Elucidating the molecular determinants of length-dependent force production is therefore essential for understanding both normal muscle physiology and disease. Because sarcomeric organization and many regulatory interactions are evolutionarily conserved, insect muscle provides a powerful experimental system for dissecting the structural and mechanical basis of mechanosensitive contraction. Asynchronous IFM is particularly valuable for studying length-dependent force production because it minimizes the confounding effects of beat-to-beat Ca^2+^ transients and repeated electrical stimulation that are characteristic of cardiac muscle. This experimental separation between neural input and mechanical oscillation enables detailed investigation of stretch-sensitive contractile mechanisms with reduced interference from excitation-contraction coupling dynamics.

## 4 Sarcomere and myofilament proteins in animal and insect muscles

To understand how muscle force production and contractile performance are achieved and regulated, it is necessary to examine the molecular architecture of the sarcomere and the proteins that comprise the myofilament. While the overall organization of the sarcomere is conserved across species, insects and vertebrates exhibit both shared and specialized myofilament components that contribute to distinct modes of contraction, including synchronous and asynchronous activity. In the following sections, we compare the structure, function, and regulation of key sarcomeric proteins in animal and insect muscles, highlighting conserved mechanisms alongside insect-specific adaptations that have shaped diverse contractile behaviors. Mutations in conserved sarcomeric proteins that have revealed key mechanisms of muscle performance are summarized in **Table 1** and organized by mechanistic class to highlight shared sarcomere-level principles.

**Table 1:** Conserved sarcomere mutations revealing mechanisms of striated muscle performance in insect models and vertebrate disease.

I. Thin-filament structural integrity and sarcomere assembly						
Sarcomere protein	Mutation / variant	Molecular / structural effect	Sarcomere-level functional impact	Insect model phenotype	Vertebrate disease association	Reference
Actin (*Act88F*)	E93K	Disrupts F-actin polymerization and Z-disc anchoring	Markedly reduced myofibrillar stiffness; failure of sarcomere assembly	Loss of Z-discs; disorganized IFM; flightless	Congenital myopathy (ACTA1-associated)	Sevdali 2013; Sparrow 1991
Actin (*Act88F*)	V163L/M	Impairs nucleotide binding; destabilizes filament structure	Reduced filament stability; compromised force transmission	Zebra bodies; IFM structural degeneration	ACTA1 myopathies	Sevdali 2012
Actin (*Act88F*)	I136M	Subtle perturbation of actin filament conformation	Mild impairment of sarcomere stability	Mild IFM structural defects; partial flight impairment	Mild nemaline myopathy variants	Sevdali 2012
Troponin T (*up^2^*)	Reduced/absent TnT protein	Loss of TnT-mediated stabilization of thin filament complex	Destabilized thin filaments; impaired sarcomere maintenance	Severe IFM disorganization; thin filament loss	Thin-filament destabilization phenotypes	Fyrberg 1990
Troponin T (*up^3^*)	Reduced/absent TnT protein	Compromised troponin-tropomyosin integration	Impaired filament integrity and assembly	IFM degeneration; loss of ordered sarcomeres	Troponin T–associated myopathies	Fyrberg 1990
Tropomyosin (*Tm2*)	E139K, V129A, A155T	Alters tropomyosin structural stability along actin filament	Impaired thin filament incorporation during development	Defective embryonic muscle morphogenesis; reduced muscle integrity	Skeletal muscle developmental myopathies	McAdow 2022
Tropomyosin (*Tm2*)	E122K	Disrupts tropomyosin:actin stabilization during early myogenesis	Abnormal filament assembly and fiber maturation	Shortened embryonic muscles; structural deficits	Congenital fiber type disproportion (CFTD)	McAdow 2022
II. Thin-filament regulatory tuning (Ca^2+^ sensitivity and cooperative activation)						
Troponin T (*up*)	*up^101^* (E88K; residue identified in later molecular analyses)	Disrupts TnT–tropomyosin regulatory coupling	Enhanced thin-filament activation and hypercontractile instability	Flightless; fiber shortening and progressive IFM degeneration	Regulatory thin-filament myopathy parallels	Fyrberg 1990; Nongthomba 2003; Viswanathan 2013
Troponin T (*up*)	*up¹* (exon 10A splicing defect)	Disrupts IFM-specific TnT isoform incorporation	Impaired thin-filament maturation and sarcomere assembly	Zebra bodies; defective IFM organization; flightless	Nemaline myopathy–like pathology	Nongthomba 2003; Fyrberg 1990
Troponin I (*wupA*)	*hdp^2^* (A116V)	Weakens inhibitory region–actin interaction	Increased activation and impaired relaxation	Loss of flight; shortened sarcomeres	Hypercontractile regulatory myopathies	Nongthomba 2004
Troponin I (*wupA*)	*hdp^3^*	Loss of functional inhibitory control	Constitutive thin-filament activation	IFM fails to form; degeneration	Contractile dysregulation phenotypes	Nongthomba 2003, 2004
Troponin C (*TpnC41C*)	IFM-specific isoform variant	Alters Ca^2+^ binding properties	Impaired activation dynamics in specialized muscle	Flightless; sarcomere disorganization	Troponin C deficiency phenotypes	Chechenova 2017
Tropomyosin (*Tm2*)	K49Del	Reduces actin affinity; shifts regulatory equilibrium	Increased thin-filament activation at submaximal Ca^2+^	Developmental contractile imbalance	Nemaline myopathy	McAdow 2022
Actin (*Act88F*)	D292V	Perturbs tropomyosin positioning along actin	Disrupted cooperative activation	Impaired IFM contraction	ACTA1-associated myopathy	Sevdali 2012
Actin (ACTC1 / cardiac α-actin)	A295S	Disrupts actomyosin interactions within actin subdomain 3	Enhanced crossbridge recruitment; prolonged force generation; impaired relaxation	Diastolic dysfunction in *Drosophila* cardiac models	Hypertrophic cardiomyopathy (HCM)	Viswanathan 2017; Viswanathan 2020
Actin (ACTC1 / cardiac α-actin)	M305L	Alters localized actin–tropomyosin interactions	Impaired thin-filament regulation; reduced diastolic volume	Abnormal cardiac muscle behavior in *Drosophila*	Hypertrophic cardiomyopathy (HCM)	Schmidt & Cammarato 2020; Huang 2024
Actin (ACTC1 / cardiac α-actin)	A331P	Destabilizes filament organization and alters tropomyosin positioning	Increased basal tension; impaired myofibrillar integrity	IFM structural instability	Hypertrophic cardiomyopathy (HCM)	Huang 2024
III. Crossbridge kinetics and power output						
Actin (*Act88F*)	E334K, E364K, R372H, E316K, G368E, V339I	Alters surface charge at actin- myosin interface	Reduced crossbridge efficiency; impaired force production	Defective flight muscle performance	Surface residue variants affecting contractility in ACTA1	Just 1993
Myosin heavy chain (*Mhc*)	E701K	Reduces ATPase activity; decreases actin-binding affinity	Decreased crossbridge cycling efficiency; reduced power output	Complete loss of flight; impaired jump performance	MYH2-associated myopathy (IBM-3 analog E706K)	Wang 2012
Myosin heavy chain (*Mhc*)	R1845W	Disrupts thick filament assembly and myosin rod stability	Reduced A-band integrity; impaired force transmission; progressive myofibrillar disorganization	Reduced flight and jumping ability; abnormal wing posture; selective A-band loss	Myosin storage myopathy (MYH7 variants)	Dahl-Halvarsson 2020; Viswanathan 2025
Myosin heavy chain (*Mhc*)	E1883K	Alters myosin tail interactions; filament destabilization	Progressive thick filament disorganization	Degenerating IFM fibers; reduced flight	Myosin storage myopathy	Viswanathan 2025
Myosin heavy chain (*Mhc*)	L1793P	Impairs filament incorporation and stability	Reduced thick filament structural integrity	IFM degeneration; decreased mobility	MYH7-related myopathy	Viswanathan 2025
Myosin heavy chain (MYH3 homolog)	F437I	Alters motor-domain mechanics and force transmission	Impaired contractile coordination during development	Developmental locomotor defects in *Drosophila*	Distal arthrogryposis type 1 (DA1)	Guo 2020
Myosin heavy chain (MYH3 homolog)	A234T	Perturbs embryonic myosin motor activity	Altered force generation during muscle development	Developmental muscle dysfunction	Distal arthrogryposis type 2B (DA2B / Sheldon-Hall syndrome)	Guo 2020
Myosin heavy chain (β-cardiac myosin)	R249Q / R249	Reduces crossbridge binding and actin-activated ATPase activity	Impaired force production despite proposed hypercontractile human phenotype	Reduced IFM contractility	Hypertrophic cardiomyopathy (HCM)	Bell 2019; Kronert 2022
Myosin heavy chain (β-cardiac myosin)	R369H / R369Q	Minimally alters ATPase activity but reduces maximal actin binding	Altered actomyosin interaction and muscle-type specific dysfunction	More severe skeletal than cardiac muscle defects in *Drosophila*	Dilated cardiomyopathy (DCM)	Trujillo 2022
Myosin heavy chain (CardM2 isoform)	CardM2 expression	Alters stretch-sensitive myosin mechanics	Increased stretch activation (SA) and shortening deactivation (SD) tension	Enhanced jump muscle oscillatory mechanics	Mechanosensitive contractile regulation	Bell 2025
IV. Electrostatic modulation and hypercontractility						
Actin (*Act57B*)	K326Q/K328Q (pseudo-acetylation)	Neutralizes conserved lysine residues at the actin surface; disrupts electrostatic interactions with tropomyosin and myosin	Constitutive thin filament activation; hypercontractile instability	Reduced climbing; progressive muscle degeneration	Nemaline myopathy; thin-filament dysregulation	Viswanathan 2015
Actin (*Act57B*)	K328Q	Removes positive charge at key actin: Tm interface	Enhanced acto-myosin interaction; impaired relaxation	Complete loss of flight; severe degeneration	Regulatory thin-filament myopathies	Viswanathan 2015
Actin (*Act57B*)	K326Q/K328Q (double mutant)	Combined charge neutralization amplifies electrostatic imbalance	Severe hypercontraction; sarcomere collapse	Strong degenerative phenotype	Hypercontractile thin-filament disorders	Viswanathan 2015

Mutations discussed in this review are organized by mechanistic class rather than by protein identity to emphasize conserved sarcomere-level principles governing muscle performance. Mechanistic categories reflect primary functional perturbations, including (I) thin-filament structural integrity and sarcomere assembly, (II) Ca^2+^-dependent regulatory tuning of thin-filament activation, (III) crossbridge kinetics and power generation, and (IV) electrostatic modulation associated with hypercontractility and degeneration. For each mutation, molecular or structural effects are distinguished from sarcomere-level functional consequences and organismal phenotypes. Vertebrate disease associations illustrate conserved pathogenic mechanisms revealed through insect models.

### 4.1 Actin

The thin-filament protein actin, one of the most highly conserved eukaryotic proteins, is a critical component of the myofilament in both insects and vertebrates (Vigoreaux, 2001; Roper et al., 2005). The most extensively characterized *Drosophila* isoform, *Actin88F*, shares about 90% sequence identity and nearly identical structural homology to vertebrate muscle actin (Roper et al., 2005). Actin88F is the only isoform expressed in *Drosophila* IFM and is not required for survival, making it especially useful to study actin *in vivo* (Hennessey et al., 1993; Haigh et al., 2010). Several historic mutational studies on the *Drosophila Actin88F* gene have documented effects on actin polymerization as well as myofibril development and stability. *Actin88F^E93K^* mutants are viable, but lack both Z-discs and sarcomeric structure, yielding muscle fibers with half the stiffness of wild-type fibers (Sparrow et al., 1991). Because *Actin88F^E93K^* mutants are flightless, the authors propose that loss of tension under activating conditions of stretch, ATP, and Ca^2+^ depends on the distance between thick and thin filaments (referred to as the filament lattice), and presence of the Z-disc, the protein-rich lateral boundary of the sarcomere (Sparrow et al., 1991; Frank and Frey, 2011). Supporting the idea that thin-filament composition is critical to the structural stability of IFM, mutations in actin that are homologous to human congenital myopathy alleles (detailed in section 6.2 and in **Table 1**) produce disrupted sarcomere architecture in *Drosophila* IFM, including incomplete Z-discs, disorganized actin filaments, and “zebra bodies”, a pathological hallmark of human nemaline myopathies (Nongthomba et al., 2007; Sevdali et al., 2013). Overexpression of these actin variants interferes with sarcomere organization in a manner strikingly similar to human disease pathology, underscoring the sensitivity of IFM thin-filament structure to perturbations in actin-associated regulatory interactions (Sevdali et al., 2013).

Using the same *Actin88F* E93K point mutation, Razzaq et al. further dissected the molecular basis underlying these structural and mechanical defects, demonstrating that E93K reduces actin-dependent cell motility *in vitro* without impairing myosin force transmission (Razzaq et al., 1999). Through stop-flow kinetics experiments using purified actin isoforms, combined with “optical tweezer” based force measurements examining interactions between purified *Drosophila* actin filaments and rabbit skeletal muscle heavy meromyosin, the authors identified E93 as a secondary myosin-binding site, revealing a previously unrecognized actomyosin interface (Razzaq et al., 1999).

### 4.2 Myosin

Myosin is the principal motor protein responsible for force generation in both vertebrate and invertebrate muscle. Although the overall architecture and ATP-dependent cross-bridge cycle are highly conserved, vertebrates and insects differ substantially in myosin isoform diversity and regulatory specialization. In vertebrates, distinct skeletal, cardiac, and smooth muscle myosin heavy chain isoforms are encoded by separate genes and contribute to muscle-type specific contractile properties (Baldwin et al., 2013). In contrast, *Drosophila* myosin heavy chain is encoded by a single alternatively spliced gene, *Mhc*. Like many other contractile proteins, extensive alternative splicing generates developmental and tissue-specific isoforms that fine-tune functional properties (Swank et al., 2000). Whereas vertebrate skeletal and cardiac muscles achieve functional specialization largely through expression of distinct myosin heavy chain genes, insects extensively diversify myosin function through alternative splicing of a single *Mhc* locus. *Mhc* can be extensively alternatively spliced to yield hundreds of distinct myosin heavy chain isoforms, up to approximately 480 in *Drosophila*, enabling a single gene to produce muscle-type specific contractile phenotypes (Dahl-Halvarsson et al., 2020).

*Mhc* deficiencies have revealed important relationships between gene copy number and muscle function. While *Mhc^1^* null flies are not viable, heterozygous *Mhc^1/+^* flies are flightless and exhibit an approximately 50% reduction in thick filaments across all muscles (O'Donnell and Bernstein, 1988). In early genetic studies, expression of headless myosin (*Mhc* transgenes expressing either the adult or embryonic rod) in both wild-type and *Mhc^10^*-null backgrounds demonstrated that the myosin head is not required for thick filament assembly. The myosin head is, however, necessary for higher-order myofibril organization, with even low levels of transgene expression severely impairing flight (Cripps et al., 1999; Swank et al., 2000).

Other *Mhc* mutations are homozygous viable but selectively affect flight or jump muscle function. In the flight muscle-specific allele *Mhc*^5^, which disrupts a residue required for ATP entry or ADP exit from the active site, myofibrils assemble normally but undergo progressive degeneration with age (Cripps et al., 1999). A second flight muscle-specific substitution, *Mhc*¹³, yields a proteolytically insensitive myosin heavy chain, resulting in hypercontracted indirect flight muscle and flightlessness (Cripps et al., 1999). This substitution alters a conserved, hydrophilic glutamic acid residue within the coiled-coil rod region of both muscle and non-muscle myosins, suggesting that maintenance of charge and hydrophobicity in this region is critical for proper myosin spacing within the thick filament *in vivo (*Kronert et al., 1995).

Beyond mutations affecting myosin assembly or stability, alternative splicing within the myosin heavy chain itself provides a powerful mechanism for tuning thick-filament architecture and muscle performance. Substitution of the fast-muscle hinge A (exon 15a) with the slow-muscle hinge B (exon 15b) in *Drosophila* indirect flight muscle lengthens the myosin rod, leading to increased A-band and sarcomere lengths through longitudinal addition of myosin heads along thick filaments. Despite its distal position relative to the catalytic domain, hinge B alters myosin kinetics, increasing apparent actin affinity without changing maximal actin-activated ATPase activity. At the fiber scale, these changes shift viscoelastic and oscillatory properties, altering work and power-frequency relationships. At the organismal level, hinge substitution reduces wing-beat frequency and flight performance, indicating that the S2/LMM hinge acts as a distal structural regulator coordinating thick-filament geometry with cross-bridge dynamics (Miller et al., 2009).

Invertebrate studies investigating myosin in the context of stretch activation dynamics are not limited to *Drosophila*. Cryoelectron tomography studies in *Lethocerus* and *Bombus* flight muscle have revealed a stretch activation mechanism in which myosin heads form weak attachments to actin and undergo azimuthal rotations around the thin filament, generating torque (Herranz et al., 2005b; Wu et al., 2010). This directed movement promotes conversion to a strongly bound, force-producing state, thereby amplifying myosin recruitment and force generation. These findings support models in which stretch activation may arise in part from intrinsic mechanical properties of the myosin motor, with the myosin head potentially functioning as a strain-responsive element (Iwamoto and Yagi, 2013; Iwamoto, 2017). Importantly, the conservation of myosin motor structure and regulatory function across species has enabled IFM models to provide mechanistic insight into vertebrate myosin-associated disease, discussed further in Section 6.3.

### 4.3 Troponin

Asynchronous, stretch-activated insect flight muscle is characterized by unique geometry and structure because of its unusually high but efficient energy demands. Thin filaments are placed midway between 2 thick filaments, whereas cardiac and skeletal muscle thin filaments are equally positioned between 3 myosin thick filaments (Ha et al., 2003). The highly ordered lattice geometry of IFM imposes distinct spatial constraints on thin-filament regulatory proteins, producing more uniform alignment of actin, tropomyosin (Tm), and the troponin (Tn) complex along the thin filament than is typically observed in vertebrate cardiac or skeletal muscle. Similarly, all troponins have the same azimuthal orientation in relation to the two neighboring myosin thick filaments (Bullard and Pastore, 2019). These features produce a level of actin and troponin periodicity in *Drosophila* IFM that is not observed in vertebrate skeletal or cardiac muscle. This high degree of structural order improves the ability to resolve dynamic actomyosin interactions using structural, diffraction, and computational approaches, making IFM a particularly powerful system for investigating conserved mechanisms of stretch-regulated contraction.

Because these structural specializations directly influence thin-filament regulation, comparative analysis of Tn-Tm complex components has provided important insight into both conserved and specialized mechanisms of stretch-activated contraction. Vertebrate and invertebrate Tn-Tm complexes are highly homologous, each playing a central role in the Ca^2+^-dependent regulation of striated muscle contraction. Troponin is a heterotrimer with three distinct subunits named according to their functions: the Ca^2+^ binding subunit troponin C (TnC), the actomyosin ATPase inhibitory subunit troponin I (TnI), and the tropomyosin binding subunit troponin T (TnT) (Greaser and Gergely, 1971; Sheng and Jin, 2016). While some thin filament proteins in invertebrates have nearly identical homology to vertebrates, other well-studied subunits have evolutionarily diverged discrete molecular functions (Herranz et al., 2005b; Herranz et al., 2005a). The following sections highlight conserved and specialized functions of Tn-Tm regulatory proteins in insect and vertebrate muscle, with emphasis on mechanisms contributing to stretch-activated contraction. Studies in invertebrates have provided important insight into evolutionarily conserved mechanisms regulating Ca^2+^ sensitivity, contractility, and energetic efficiency.

#### 4.3.1 Troponin C (TnC)

The Ca^2+^-binding subunit of troponin, TnC, belongs to the calmodulin superfamily of genes (Collins, 1991). TnC has a characteristic dumbbell structure with two globular Ca^2+^-binding EF hand domains connected by an alpha-helix (Vassylyev et al., 1998). Vertebrate TnC is characterized by a conserved C-terminal domain containing 2 high affinity metal-ion binding sites (sites III and IV) occupied by Mg^2+^ at rest and by Ca^2+^ during activation of contraction. In mammals, troponin C (TnC) is encoded by two genes that differ in their variable N-terminal regions, which tune Ca^2+^ sensitivity through interactions with troponin I (TnI) (Herranz et al., 2005b). In fast skeletal muscle, the N-terminal domain of TnC contains two low-affinity metal-ion binding sites (sites I and II) that regulate contraction, whereas slow/cardiac TnC retains only a single functional regulatory site (site II). During activation, increased intracellular Ca^2+^ promotes Ca^2+^ binding to the N-terminal globular domain of TnC, triggering conformational changes that initiate contraction (Singh et al., 2014).

In invertebrates, TnC is encoded by many genes. *C. elegans* TnC is encoded by two genes, *Lethocerus* by three, *Drosophila* and *Apis* TnC encoded by five, and *Anopheles* by six transcripts (Qiu et al., 2003; Herranz et al., 2005b). The defining conserved feature of TnC is the repeated EF-hand motif, while differences in cation-binding properties and expression patterns contribute to functional specialization across muscle types (Herranz et al., 2005b). These mechanisms have been particularly well studied in *Drosophila* because of its powerful genetic tools (Chechenova et al., 2015; Chechenova et al., 2017), and in *Lethocerus* because of its relatively large flight muscles. Both *Drosophila* and *Lethocerus* asynchronously contract their IFM in response to periodic stretch at permissive Ca^2+^ concentrations. The IFM of *Drosophila* and *Lethocerus* contains a unique TnC with a single C-terminal Ca^2+^-binding site (site IV). IFM also contains a minor TnC isoform with an additional low-affinity N-terminal Ca^2+^-binding site (site II). Functional specialization of IFM TnC isoforms contributes directly to stretch-activated contraction. Initial activation of IFM acts through the two-site TnC isoform, whereas full stretch-dependent activation is mediated primarily through the unique one-site TnC (Qiu et al., 2003). Crucially, without this site, stretch activation is not observed (Agianian et al., 2004).

Recombinant genetic studies in *Lethocerus* have identified different isoforms of TnC within single myofibrils. The F1 TnC isoform has a single high-affinity C-terminal Ca^2+^-binding site which generates stretch-activated tension. The F2 TnC isoform has one high-affinity C-terminal Ca^2+^-binding site and one exchangeable low-affinity N-terminal Ca^2+^-binding site and generates isometric contraction (Agianian et al., 2004). Studies in *Lethocerus* demonstrate that TnC isoform expression defines IFM function and that spatially restricted expression can reversibly specify functional identity within the same myofibril. Notably, the two TnC isoforms regulate a shared thin filament through distinct, independent mechanisms. Comparable specialization occurs in vertebrate muscle, where differential expression of TnC isoforms contributes to functional differences between skeletal and cardiac thin filaments. For example, cardiac TnC is less inhibited than skeletal muscle TnC at low intracellular Ca^2+^ concentrations (Maytum et al., 1999).

#### 4.3.2 Troponin I (TnI)

The inhibitory subunit of the Tn complex, troponin I (TnI) has 3 vertebrate isoforms (Chong and Jin, 2009). In low cytoplasmic Ca^2+^, TnI binds actin and inhibits actomyosin ATPase (Farah et al., 1994). When intracellular Ca^2+^ increases, the C-terminus of TnC binds TnI, releasing it from actin and initiating contraction (Farah et al., 1994). The 3 vertebrate TnI isoforms are differentially expressed in fast twitch, slow twitch, and cardiac muscle, and isoform expression is modified during fiber-type switching, development, and the progression of various disease pathologies (Sheng and Jin, 2014).

Insects encode a single troponin I (TnI) gene. In *Drosophila*, this gene comprises 13 exons and is alternatively spliced to produce at least 10 isoforms (Herranz et al., 2005a). Mutational analyses of indirect flight muscle- and jump-muscle specific isoforms have demonstrated that TnI-dependent disinhibition of contraction is required for normal myofibril development (Nongthomba et al., 2004). Regulation of TnI splicing is mediated in part by the RNA-binding protein Rbfox1, which promotes adult muscle diversity in *Drosophila* through fiber type-specific gene expression and alternative splicing. Disruption of this regulatory pathway, particularly loss of an indirect flight muscle-specific TnI isoform required for proper myosin regulation, results in severe structural defects in indirect flight muscle (Nikonova et al., 2022). Specifically, mutations in *Drosophila* TnI that result in hypercontraction during development manifest in aberrations in muscle fiber morphology (Nongthomba et al., 2003; Nongthomba et al., 2004). Dysregulation of *Drosophila* TnI leads to concurrent reduction of other thin filament regulatory complex proteins, including TnT and flight-muscle specific Actin88F (Nongthomba et al., 2004). Transcriptional and translational regulation of contractile regulatory elements is not limited to *Drosophila* and suggests critical stoichiometric relationships between Tn-Tm complex proteins.

*Lethocerus* troponin I contains an unusually large flight muscle-specific C-terminal proline-alanine-rich extension, a specialization absent from vertebrate TnI isoforms but shared with several other insect flight muscle thin-filament proteins (Herranz et al., 2005a; Mateos et al., 2006). This Pro-Ala-rich extension substantially increases the molecular mass of IFM-specific TnI (approximately 70–80 kDa compared with ~25 kDa for non-IFM TnI), and the protein is therefore commonly referred to as troponin H (TnH) or heavy troponin (Iwamoto, 2013a). X-ray diffraction studies show that heavy Tn takes part in, but is not required for stretch activation (Iwamoto, 2013a). Further analysis suggests that the Pro-Ala-rich extension helps maintain the highly ordered lattice architecture characteristic of invertebrate IFM. Enzymatic removal of this region disrupts IFM-specific filament organization and produces structural behavior more similar to vertebrate striated muscle, indicating that the extension contributes to the specialized lattice geometry required for oscillatory contraction (Iwamoto, 2013a; Iwamoto, 2013b).

Stretch-sensitive regulation of contraction is not unique to insect flight muscle and has also been implicated in vertebrate cardiac physiology, including mechanisms contributing to length-dependent activation and the Frank-Starling response (de Tombe et al., 2010; Iwamoto, 2013a). Comparative studies of insect and vertebrate TnI therefore provide an opportunity to distinguish conserved regulatory principles from IFM-specific specializations. In many invertebrate model systems, selective removal or modification of insect-specific protein sequences has helped identify structural features required for oscillatory contraction while also revealing regulatory mechanisms shared across striated muscle types. Invertebrate models further permit analysis of TnI mutations that are lethal in vertebrate systems, providing insight into how coordinated thin-filament regulation contributes to muscle dysfunction and disease progression (Huang et al., 1999).

#### 4.3.3 Troponin T (TnT)

The tropomyosin-binding subunit of troponin, TnT exists as three developmentally regulated, muscle-type specific isoforms in vertebrates (Sheng and Jin, 2014). TnT comprises two functional domains: the N-terminal T1 region, which binds tropomyosin, and the C-terminal T2 region, which contains binding sites for TnC, TnI, and F-actin (Perry, 1998) as well as an additional tropomyosin binding site (Jin and Chong, 2010). The N-terminus of TnT is “hypervariable”, having variable lengths and amino acid sequences (Perry, 1998). The hypervariable N-terminal region does not bind other myofilament proteins, but rather shows a range of metal ion-binding affinities. Fluorescence spectral analyses and protein binding studies support a mechanism in which the N-terminal variable region of TnT modulates conformation to specifically modify muscle contractility (Wang and Jin, 1998; Biesiadecki et al., 2007; Jin et al., 2008; Wei and Jin, 2011).

The N-terminal variable region of TnT from avian pectoral muscle has a long glutamic acid (Glu)-rich segment of unknown function (Zhang et al., 2011). Interestingly, the invertebrate isoform of TnT specific to IFM also has a C-terminal Glu-rich extension (Fyrberg et al., 1990). Sequence conservation between muscles of diverse flying species could indicate powerful functional conservation since the N-terminal variable region of TnT is known to fine-tune muscle contractility and efficiency. In line with this concept, experiments in *Drosophila* show that the Glu-rich long C-terminal extension of IFM-specific troponin T is required to achieve normal muscle performance. The loss or truncation of this extension leads to impaired flight capacity, impaired myofibril organization and a changed thin-filament lattice spacing, which highlights the importance of this feature in fine-tuning sarcomere structure to stretch-activated contraction (Cao et al., 2020).

*Drosophila* TnT is encoded by a single, alternatively spliced gene (*upheld, up*) containing 11 exons, generating muscle-type specific isoforms. Among these, isoform 10A is the best characterized and is essential for asynchronous flight muscle contraction (Nongthomba et al., 2007). Exons 10A and 10B are mutually exclusive: exon 10A is expressed in the IFM and the tergal depressor of the trochanter (TDT), whereas exon 10B is expressed in all other larval and adult striated muscles (Herranz et al., 2005a). The IFM-specific TnT isoform is highly conserved across *Drosophila* species and among flying insects, exhibiting strong sequence and functional homology. A distinctive polyglutamate-rich C-terminal extension, unique to insect IFM TnT, is thought to promote thickening of the TnT filament lattice to support high-frequency oscillatory contractions (Benoist et al., 1998; Domingo et al., 1998). Consistent with this model, the *up¹* mutation disrupts exon 10A splicing, abolishes IFM-specific TnT expression, and leads to severe myofibrillar disorganization in adult flight muscle (Nongthomba et al., 2007).

Precise regulation of this alternative splicing event is critical for muscle development and integrity. Muscleblind (Mbl), the *Drosophila* ortholog of human Muscleblind-like 1 (MBNL1), directly regulates inclusion of exon 10A in the IFM. Loss-of-function *mbl* mutants exhibit defects in Z-band organization and muscle attachment, while *mbl* overexpression induces spatially restricted apoptosis through interactions with apoptotic regulators (Vicente-Crespo et al., 2008). These findings establish a mechanistic link between splicing control of TnT and the structural integrity of muscle tissue.

Consistent with the essential role of isoform 10A, both homozygous and heterozygous *up* mutants with defects in exon 10A splicing are flightless (Nongthomba et al., 2007). Dominant TnT mutations were originally identified by the characteristic “upheld” wing posture of IFM mutant flies, although age-dependent impairments in jumping have also been reported (Nongthomba et al., 2007). Because IFM and TDT expression is restricted to adults, larval crawling and adult walking behaviors remain unaffected.

This work also provided early evidence for developmentally regulated isoform switching in insect muscle. In contrast to deuterostomes, protostomes undergo distinct larval, pupal, and adult muscle programs during metamorphosis. Accordingly, the single *Drosophila* TnT gene expresses isoform 10B during larval development before switching to the highly specialized 10A isoform in adult IFM to accommodate flight and stretch-activated contraction. Comparable developmental isoform switching also occurs in vertebrate muscle, including cardiac TnT (Cooper, 1998). Although vertebrates encode multiple tissue-specific TnT genes (Wei and Jin, 2016), the core tropomyosin- and TnC-binding domains regulating Ca^2+^-dependent contraction remain highly conserved (Nongthomba et al., 2007). Importantly, mutations affecting IFM-specific exon 10A disrupt sarcomere organization and produce pathological features resembling human nemaline myopathies, including zebra bodies (Nongthomba et al., 2007). Taken together, these findings demonstrate how alternative splicing of TnT contributes to muscle-type specific contractile specialization while preserving core regulatory mechanisms across species. In this context, invertebrate models provide a valuable system for dissecting how TnT isoform regulation influences muscle development, contractile function, and disease pathology.

### 4.4 Tropomyosin

Stretch activation in insects is based on the theory of oscillating muscle contraction: each time a muscle contracts, it stretches an opposing muscle which subsequently contracts, stretching an opposing muscle, and so on and so forth (Holmes, 2011). The system is inhibited if myosin cannot bind actin filaments, which in striated muscle is sterically inhibited by tropomyosin (Tm) molecules. X-ray diffraction studies in *Lethocerus* establish that in insect flight muscle, both Ca^2+^ and stretch are required for the remarkably rapid and efficient steric blocking and unblocking of actin binding sites by Tm (Pringle, 1978). Stretch mechanically pulls Tm away from actin binding sites, accommodating extremely high wing beat frequencies at priming Ca^2+^ levels. This allows rapid contraction and relaxation asynchronous with action potentials (Pringle, 1978).

In vertebrate skeletal and cardiac muscle, tropomyosin similarly regulates access to myosin-binding sites on actin through Ca^2+^-dependent interactions with the troponin complex (Matyushenko et al., 2017). Vertebrate tropomyosin isoforms contribute to muscle-type specific differences in contractility, Ca^2+^ sensitivity, and thin-filament stiffness, particularly in cardiac muscle where altered tropomyosin regulation has been linked to cardiomyopathy and impaired contractile performance (Bai et al., 2013). Although vertebrate muscles do not exhibit the extreme oscillatory behavior characteristic of asynchronous IFM, many of the underlying steric regulatory principles governing actomyosin activation are conserved across systems.

In *Drosophila*, two IFM-specific heavy Tm isoforms were discovered that are important for stretch activation. *Drosophila* Tm is encoded by two genes, *Tm1* and *Tm2*, that are alternatively spliced into either the standard muscle isoform, Tm 128, or the IFM-specific heavy isoforms, TH-33 and TmH-34 (Mateos et al., 2006). These muscle isoforms are part of a three-gene cluster: *Tm1* and *Tm2* encode muscle specific tropomyosins, while the closely linked *Tm3* gene encodes a cytoplasmic, non-muscle tropomyosin isoform (Bautch et al., 1982). The proximity of these genes and the divergence of their expression patterns suggest an evolutionary relationship between them (Bautch et al., 1982). During stretch activation, these large IFM-specific homo or heterodimers can extend filament geometry out-of-phase or, alternatively, act as stretch sensors themselves (Mateos et al., 2006). The C-terminal extension is rich in Pro-Ala-Gly-Glu and is not unique to *Drosophila*; a similar region is found in *Lethocerus* flight muscle (and discussed above in section 4.2.3) (Bullard et al., 1988). Evolutionary conservation of the amino acid sequence in other holometabolous insects (those that undergo complete metamorphosis) such as *Apis* and *Anopheles* indicate a structure-function relationship that warrants further study (Herranz et al., 2005a; Mateos et al., 2006).

While alternative splicing of thin-filament regulatory proteins such as TnT provides a powerful mechanism for specifying muscle-type-specific contractile behavior, splicing alone cannot account for the extreme mechanical demands of asynchronous flight muscle. In these muscles, precise control of sarcomere stiffness and elastic recoil is equally critical for maintaining structural integrity and sustaining high-frequency oscillatory contractions. Insects achieve this through a specialized network of elastic proteins that complement thin-filament regulation, collectively tuning passive tension, filament spacing, and force transmission within the sarcomere.

## 5 Elastic proteins in striated muscle

### 5.1 Titin and length-dependent activation

Asynchronous flight muscle sarcomeres are compact, with thick filaments positioned close to the Z-disc and short, relatively inelastic I-bands (Bullard et al., 2005). In vertebrate striated muscle, compliance and passive tension are largely governed by the giant elastic protein titin, which spans from the Z-disc to the M-line (Bullard et al., 2005). Titin contributes to length-dependent regulation of force production through modulation of interfilament lattice spacing, as well as through alternative splicing and post-translational modifications that tune its stiffness (Fukuda and Granzier, 2005). In the vertebrate heart, titin functions as a primary determinant of passive stiffness and physiological elasticity. Distinct regions, including the N2B, PEVK, and tandem Ig domains, differentially contribute to myocardial compliance through alternative splicing and post-translational modification (Loescher et al., 2023; Rees et al., 2023). Consistent with this central role, truncating variants in titin increase susceptibility to pacing-induced arrhythmia, disrupt sarcomere organization, and reduce contractile force in atrial induced pluripotent stem cell-derived cardiomyocytes (Huang et al., 2025). Although insects lack a single titin ortholog spanning the full sarcomere, analogous elastic functions are distributed across modular proteins encoded by the titin homolog *sallimus* (*sls*) (Bullard et al., 2005; Lindstedt and Nishikawa, 2017).

Alternative splicing of *sls* generates multiple elastic isoforms, including kettin, which is prominently expressed in flight muscle (Kolmerer et al., 2000; MaChado and Andrew, 2000). Like vertebrate titin, *Drosophila* Sls proteins contain immunoglobulin (Ig), PEVK, and fibronectin (Fn) domains, reflecting a conserved architectural logic underlying sarcomere elasticity (Fukuda et al., 2008). Beyond muscle, *Drosophila* titin localizes to spindle fibers in crane-fly and locust spermatocytes, where it associates with microtubules, actin, myosin, and spindle-matrix proteins, suggesting a broader role as an elastic scaffold in both sarcomeric and non-sarcomeric assemblies (Fabian et al., 2007).

### 5.2 Kettin and passive stiffness of flight muscle

Kettin is conserved across nematodes and arthropods and exhibits high sequence conservation (Lakey et al., 1993; Kolmerer et al., 2000). In IFM, kettin binds actin near the Z-disc, reinforcing thin filaments and contributing substantially to passive stiffness. The N-terminus of kettin is embedded in the Z-disc, and because the IFM I-band is only ~50 nm long, the C-terminus reaches the end of the A-band (van Straaten et al., 1999). *Drosophila* flight muscles are notably stiff and relatively inextensible, a property largely conferred by kettin (Kulke et al., 2001). The Ig domains of kettin anchor at the end of the A-band, and its association with actin is critical for maintaining passive tension and structural integrity during oscillatory contractions (Bullard et al., 2005).

### 5.3 Projectin and tunable elasticity

In vertebrate muscle, titin is the third most abundant sarcomeric protein and spans half the sarcomere (Lalande et al., 2017). In contrast, elastic tension in insect IFM is maintained by *sls* isoforms in combination with smaller elastic proteins, most notably projectin (Bullard et al., 2005). Projectin is a high-molecular weight protein with a large elastic region that includes a PEVK-like domain and a series of Ig domains spanning the I-band (Ayme-Southgate et al., 2005). The protein extends from the Z-disc to the borders of the A-band and contains a kinase domain near its C-terminus, analogous to the A-band region of vertebrate titin (Southgate and Ayme-Southgate, 2001).

Heterozygous projectin mutants in *Drosophila* retain normal flight ability but exhibit reduced stretch activation and impaired oscillatory contractions, suggesting that projectin contributes to residual force enhancement following active muscle lengthening (discussed in a later section) (Moore et al., 1999). The PEVK-like domain of projectin is thought to extend under stretch, increasing extensibility and protecting thin filaments from breakage in elongated sarcomeres (Bullard et al., 2005). Immunolabeling and biomechanical studies indicate that projectin binds thick filaments and cooperates with kettin to mechanically couple thin and thick filaments within the Z-disc region. Although projectin does not directly bind actin, its elastic properties contribute substantially to sarcomere resilience (Kulke et al., 2001). Thus, kettin and projectin cooperate to establish the passive stiffness required for stretch activation in *Drosophila* IFM.

The overall domain architecture of projectin is highly conserved across insects, particularly the repeated Ig and FnIII modules that support its scaffold and ruler functions (Bullard et al., 2005). By contrast, the PEVK region is sequence divergent while maintaining conserved length and unusual amino acid composition, consistent with an unstructured elastic segment. The *Drosophila* N-terminus contains 14 Ig domains divided into N8Ig and N6Ig blocks separated by the PEVK, while the core comprises repeated [Fn-Fn-Ig] modules. Conserved motifs within these regions support a model in which elastic tuning is achieved by modulating PEVK properties while preserving a rigid Ig/FnIII framework (Ayme-Southgate et al., 2008). Notably, differences in sarcomere extensibility between *Drosophila* IFM, *Drosophila* leg muscle, and *Lethocerus* IFM correlate with distinct kettin and projectin isoform compositions, reinforcing the idea that elastic protein diversity underlies muscle type specific mechanics (Kulke et al., 2001).

### 5.4 Ubiquitinated Act88F (Arthrin) and IFM specialization

In addition to elastic scaffolding, insect flight muscles exhibit specialized thin-filament composition through incorporation of ubiquitinated Act88F, historically termed arthrin, in fibrillar IFM. Arthrin was originally identified as a heavy form of actin and later shown to be a stable, mono-ubiquitinated variant of actin in which ubiquitin is covalently attached at Lys118 (Bullard et al., 1985; Burgess et al., 2004). Three-dimensional reconstructions revealed that ubiquitinated Act88F is positioned along thin filaments at a 1:7 ratio relative to actin. The ubiquitin moiety lies on the surface of actin opposite the myosin-binding interface, suggesting that this post translational modification does not obstruct cross-bridge formation but may instead influence thin-filament packing or stability (Burgess et al., 2004).

Biochemical studies further demonstrated that ubiquitinated Act88F derives directly from the IFM-specific actin isoform Act88F. In the flightless *Act88F^E93K^* mutant, both actin III and the ubiquitinated species exhibit coordinated shifts in isoelectric point, indicating a shared molecular origin (Ball et al., 1987). Formation of the modified species occurs several hours after actin synthesis, indicating that ubiquitination follows filament assembly during myofibrillogenesis rather than occurring co-translationally (Ball et al., 1987). Importantly, ubiquitinated Act88F remains stably modified, supporting a non-proteolytic structural role for ubiquitin in contractile protein regulation *in vivo (*Burgess et al., 2004).

Phylogenetic analyses across 83 insect taxa indicate that ubiquitinated Act88F evolved independently in *Diptera* and *Hemiptera*, indicating convergent evolution of this modification in asynchronous flight muscle (Schmitz et al., 2003). Although restricted to asynchronous flight muscle, its abundance does not correlate with flight mechanism, wingbeat frequency, or body size, suggesting ubiquitination is not required for stretch activation itself. Instead, this modification may fine-tune filament mechanics or structural stability under the extreme oscillatory demands of flight (Schmitz et al., 2003).

These studies demonstrate how elastic proteins and thin-filament specializations cooperate to tune passive stiffness, force transmission, and length-dependent mechanical behavior in insect flight muscle. Many of these emergent properties, including regulation of force with muscle length, force enhancement following stretch, and maintenance of sarcomere integrity during repeated contraction, are shared with vertebrate skeletal and cardiac muscle and rely on homologous molecular systems. Importantly, asynchronous IFM provides a unique experimental framework for dissecting these mechanisms because oscillatory contraction can be studied largely independently of rapid excitation-contraction coupling dynamics. Combined with powerful genetic manipulation, controlled mechanical perturbation, and high-resolution structural and physiological measurements, invertebrate muscle systems permit direct mechanistic testing of how specific proteins, splice variants, and regulatory pathways influence emergent contractile behavior. These advantages make IFM particularly valuable for linking molecular structure to muscle performance, adaptation, and disease.

## 6 Invertebrate myopathy models

Genetic myopathies encompass a diverse group of disorders arising from mutations that disrupt sarcomere assembly, contractile regulation, force transmission, or muscle maintenance (D'Amico and Bertini, 2008). Although clinically heterogeneous, many myopathies converge on defects in highly conserved structural and regulatory components of the myofilament system (D'Amico and Bertini, 2008). In this context, *Drosophila melanogaster* has emerged as a powerful experimental model for dissecting disease mechanisms, owing to the high conservation of muscle proteins, the genetic tractability of the system, and the ability to directly link molecular perturbations to muscle structure and function *in vivo* (Victor Atoki et al., 2025).

Approximately 75% of genes associated with human disease have identifiable homologs in *Drosophila* (Kreipke et al., 2017; Poovathumkadavil and Jagla, 2020; Victor Atoki et al., 2025). In addition, somatic fly muscles share key architectural features with mammalian skeletal muscle, including striated, multinucleated fibers organized into highly ordered sarcomeres (Kreipke et al., 2017; Poovathumkadavil and Jagla, 2020; Victor Atoki et al., 2025). The IFM, in particular, provide a sensitive readout of defects in sarcomere assembly, thin- and thick-filament regulation, and mechanical performance. When homologous mutations are introduced into *Drosophila* IFM and jump muscles, ultrastructural hallmarks of human disease are replicated; as a result, *Drosophila* models have been widely applied to investigate congenital myopathies arising from defects in thin-filament regulation, force generation, and sarcomere organization (Nongthomba et al., 2007; Haigh et al., 2010; Viswanathan et al., 2017). We discuss several of these mutations in detail in the following sections and in **Table 1**.

### 6.1 Congenital myopathies and thin-filament dysfunction

Congenital myopathies (CMs) are a heterogeneous group of inherited disorders caused by genetic abnormalities that disrupt the development, structure, and function of skeletal muscle fibers (Jungbluth et al., 2018). These disorders typically manifest during early development and are characterized by a slow- or non-progressive course with minimal muscle fiber degeneration, necrosis, or regeneration (Zhang et al., 2024). CMs are primarily classified into 4 clinical phenotypes based on histopathology: nemaline myopathy, core myopathy, myotubular/centronuclear myopathy, and congenital fiber-type disproportion myopathy (North et al., 2014). However, variants in the same gene may produce distinct clinical phenotypes, whereas an identical clinical phenotype can arise from mutations in multiple different genes (Lehtokari et al., 2014; Sewry and Wallgren-Pettersson, 2017; Pelin and Wallgren-Pettersson, 2019; Phadke, 2019). This pronounced genetic and clinical heterogeneity makes their comprehensive characterization particularly challenging. In this review, we focus on CM-associated dysfunction of proteins essential for skeletal muscle development and contractile function, including actin, myosin, troponin, and tropomyosin. Notably, numerous studies in *Drosophila* models have successfully recapitulated key human congenital myopathy phenotypes through targeted mutations in these proteins. *Drosophila* IFM has provided a particularly good system to study muscle function, because of its tractable genetics and its unique myofilament structure that is regulated by both stretch and Ca^2+^-dependent activation mechanisms. Invertebrate models have thereby allowed disease-associated mutations to be evaluated in a mechanically demanding context that reveals subtle defects in myofilament regulation.

### 6.2 Actin myopathies

Since the first report of ACTA1 mutations in patients with nemaline myopathy by Nowak et al. in 1999 (Nowak et al., 1999; Ohlsson et al., 2004), more than 250 pathogenic variants have been identified in this gene. These variants are most commonly associated with nemaline myopathy but have also been linked to congenital fiber-type disproportion myopathy, a disorder characterized by selective reduction in the diameter of one muscle fiber type without overt structural degeneration (Laing et al., 2009; Malfatti and Romero, 2016; Rynkiewicz et al., 2017; Labasse et al., 2022; Clayton et al., 2024). This number is expected to increase with the continued expansion and application of next-generation sequencing technologies. This heterogenicity reflects the abundant and multifunctional role of α-actin in muscle biology. Beyond serving as the principal structural component of thin filaments and mediating ATP-dependent force generation, skeletal α-actin regulates sarcomere assembly, filament dynamics, and muscle contraction through interactions with diverse actin-binding proteins such as myosin, tropomyosin, troponin, and nebulin (Ebashi, 1974; North and Laing, 2008; Nowak et al., 2013; Joureau et al., 2018). Accordingly, pathogenic ACTA1 mutations disrupt multiple actin-dependent processes and lead to a broad and clinically diverse spectrum of congenital myopathy.

As previously discussed, the functional consequences of actin mutations, particularly those affecting the Act88F isoform in IFM, have been extensively studied in *Drosophila*. Notably, analyses of *Act88F* mutations stratified by phenotypic severity have demonstrated a strong correlation between the defects observed in *Drosophila* and the clinical manifestations seen in patients harboring ACTA1 mutations (Sevdali et al., 2013). For instance, the I136M mutation is associated with relatively mild nemaline myopathy in humans (Ilkovski et al., 2001) and correspondingly does not fully abolish flight in *Drosophila*, whereas the V163L/M mutations cause complete flightlessness and severe sarcomere disorganization in flies (Sevdali et al., 2013), consistent with the more severe clinical intranuclear rod myopathy reported in human patients (Sparrow et al., 2003; Kaimaktchiev et al., 2006; Domazetovska et al., 2007).

Consistent with these structural defects, pathogenic actin variants lead to flightlessness in *Drosophila*, providing a functional correlate of the muscle weakness characteristic of human congenital myopathies. Other pathogenic actin variants produce disease through altered thin-filament regulation rather than overt sarcomeric disorganization. D292V, for example is associated with congenital fiber-type disproportion. Mechanistically, D292V strengthens actin-tropomyosin interactions, trapping the thin-filament regulatory switch in an inhibited (off) state and impairing Ca^2+^-dependent contraction. This functional defect prevents calcium-dependent muscle contraction even when signaled, resulting in significant weakness and flightlessness without the severe myofibrillar disorganization observed in other myopathic actin variants in either human muscle or homologous *Drosophila* IFM models (Clarke et al., 2007; Sevdali et al., 2013; Rynkiewicz et al., 2017).

Beyond ACTA1 variants, cardiac muscle α-actin (ACTC1) mutations are linked to human hypertrophic cardiomyopathy (HCM) (Dellefave et al., 2009; Despond and Dawson, 2018). HCM is an inherited cardiac disorder caused by mutations in sarcomeric proteins that lead to thickening of the left ventricular myocardium and impaired cardiac relaxation (Prinz et al., 2011; Baxi et al., 2016). Despond et al. classified ACTC1 mutations into mechanistic groups based on the location of the affected residue and its predicted effects on actomyosin interactions (Despond and Dawson, 2018). Introduction of human ACTC1 mutations into *Drosophila* has revealed conserved biomechanical consequences of disease-associated actin variants. Collectively, these studies demonstrate that distinct actin mutations perturb muscle function through separable effects on crossbridge recruitment, thin-filament stability, and tropomyosin positioning. For example, the A295S mutation disrupts actomyosin interactions within subdomain 3 of actin, enhancing crossbridge recruitment, prolonging systolic-like contraction, and producing diastolic dysfunction (Viswanathan et al., 2017; Viswanathan et al., 2020). In contrast, the M305L mutation alters localized actin-tropomyosin interactions and is associated with impaired muscle behavior and reduced diastolic volume in *Drosophila* cardiac models (Schmidt and Cammarato, 2020; Huang et al., 2024). IFM studies further suggest that the A331P mutation destabilizes filament organization and alters tropomyosin positioning, increasing basal tension and impairing myofibrillar integrity (Huang et al., 2024). Together, these findings indicate that distinct alterations in actin structural dynamics and regulatory protein interactions can drive diverse hypertrophic cardiomyopathy phenotypes.

While *Drosophila* is a critical tool for understanding myopathies, current models are largely limited to assessing structural and gross functional outcomes and do not fully capture fiber-type specificity or developmental stage dependent effects seen in patients. Future work combining *Drosophila* genetic models with calcium-sensitization assays and pharmacological screening platforms may enable the identification of targeted therapies that restore thin-filament regulation or contractile activation in actin-related congenital and cardiac myopathies.

### 6.3 Myosin myopathies

Whereas actin myopathies primarily disrupt thin-filament assembly and regulation, myosin myopathies arise from defects in force generation, ATPase activity, actin binding, and thick-filament stability. Most pathogenic myosin variants are missense mutations that alter one or more of these processes (Moore et al., 2012). *Drosophila* models have therefore been widely used to examine how distinct perturbations in myosin motor function contribute to muscle disease. For example, substitution of residue R249 within the myosin motor domain reduces crossbridge binding and actin-activated ATPase activity (Bell et al., 2019). Interestingly, this reduction in IFM contractility contrasts with the hypercontractile phenotype proposed for the corresponding human R249Q mutation, in which increased myosin availability may enhance actin interactions and ATPase activity (Bell et al., 2019; Kronert et al., 2022). In contrast, the R369H mutation minimally affects ATPase activity but reduces maximal actin binding (Trujillo et al., 2022). In *Drosophila*, this mutation produces more severe defects in skeletal muscle than in cardiac muscle, whereas mutation of the same residue in humans (R369Q) is associated with dilated cardiomyopathy (Trujillo et al., 2022). These findings illustrate how *Drosophila* models can distinguish between mutations that primarily affect enzymatic activity, actin binding, or force transmission despite producing overlapping disease phenotypes.

While these studies highlight the effects of motor-domain mutations on force production and actomyosin interactions, pathogenic variants can also disrupt myosin folding, filament assembly, and sarcomere stability. Inclusion body myopathy type 3 (IBM-3), for example, arises from a missense mutation (E706K) in the myosin heavy chain IIa gene (Wang et al., 2012). Introduction of the homologous E706K mutation into *Drosophila* myosin produces severe impairments in flight and jumping ability, accompanied by a tendency for mutant myosin heads to collapse and aggregate (Wang et al., 2012). These structural abnormalities are consistent with the cytoplasmic inclusions and disorganized muscle fibers observed in affected patients.

While motor-domain mutations primarily alter force production and actomyosin interactions, pathogenic variants can also disrupt myosin folding, filament assembly, and sarcomere stability. Inclusion body myopathy type 3 (IBM-3), for example, arises from a missense mutation (E706K) in the myosin heavy chain IIa gene (Wang et al., 2012). Introduction of the homologous E706K mutation into *Drosophila* myosin produces severe impairments in flight and jumping ability, accompanied by a tendency for mutant myosin heads to collapse and aggregate (Wang et al., 2012). These structural abnormalities are consistent with the cytoplasmic inclusions and disorganized muscle fibers observed in affected patients.

Beyond disorders associated with impaired force production and filament stability, pathogenic myosin variants also contribute to congenital contracture syndromes. Mutations in *MYH3*, which encodes embryonic and fetal skeletal muscle myosin, lead to disorders including Freeman-Sheldon syndrome (FSS; distal arthrogryposis type 2A, DA2A) and Sheldon-Hall syndrome (SHS; DA2B) (Toydemir et al., 2006; Beck et al., 2013; Beck et al., 2014; Agarwal et al., 2020). These conditions are characterized by multiple joint contractures collectively termed distal arthrogryposis (DA) (Desai et al., 2020). Recently, the Bernstein Laboratory generated the first *Drosophila* models of DA1 (F437I) and DA2B (A234T) (Guo et al., 2020). These models demonstrated that myosin mutations impair muscle structure and function, with the DA1 mutation specifically causing slower crossbridge detachment and increased muscle stiffness (Guo et al., 2020). Together, these studies identified potential mechanistic and therapeutic targets while establishing a tractable platform for investigating MYH3-related myopathies.

In addition to developmental myopathies, *Drosophila* has been used to model pathogenic variants in *MYH7*, which encodes the β-cardiac/slow skeletal myosin heavy chain and is a major contributor to inherited cardiomyopathies. Disease-associated variants including R1845W, L1793P, E1883K, and H1901L have been successfully modeled in flies (Viswanathan et al., 2025). Expression of the R1845W, L1793P, or E1883K variants reduces flight and jumping performance, reflecting impaired muscle function (Viswanathan et al., 2025). Ultrastructural analyses revealed shortened, thickened filaments and progressive disruption of IFM organization, indicating defects in thick-filament assembly and maintenance. Independent studies of the R1845W mutation further reported progressive myofibrillar disorganization, selective loss of A-band structures, organelle abnormalities, and thick-filament depletion (Wang et al., 2012; Trujillo et al., 2022; Madrigal et al., 2025).

Despite the absence of tissue-specific cardiac myosin isoforms, *Drosophila* models have provided important insight into the molecular mechanisms underlying human myosin myopathies. Studies spanning motor-domain mutations, inclusion body myopathy, distal arthrogryposis, and cardiomyopathy-associated variants demonstrate that distinct alterations in myosin function can impair force production, disrupt filament assembly, alter crossbridge kinetics, and compromise sarcomere stability. By linking specific molecular defects to conserved structural and functional phenotypes, *Drosophila* continues to serve as a powerful system for dissecting the pathogenic mechanisms of myosin-associated disease and identifying potential therapeutic targets.

### 6.4 Troponin myopathies

Early genetic studies in *Drosophila* demonstrated that TnI and TnT are essential for actomyosin-generated force during muscle development, based on epistasis experiments in which headless myosin mutants suppressed IFM hypercontraction (Nongthomba et al., 2003). These studies suggested that absence of *TnI* or *TnT* primarily disrupts thin filament regulatory mechanisms required early in IFM development (Nongthomba et al., 2003; Nongthomba et al., 2004; Nongthomba et al., 2007). Importantly, suppression of hypercontraction is dose dependent. Human myopathies that are linked to thin-filament contractile proteins in both skeletal and cardiac muscle are often heritable and variably penetrant (Coonar and McKenna, 1997). Genetic studies in *Drosophila* focusing on conserved sarcomeric proteins may therefore reveal important interactions underlying the human disease mechanisms.

Troponin T mutations in *Drosophila* serve as informative models for human myopathies, with several IFM-specific alleles identified. As previously discussed, *Drosophila upheld* (*up*) is a single gene that generates multiple troponin T isoforms through alternative splicing (Fyrberg et al., 1990; Viswanathan et al., 2014). Although all *up* mutants exhibit behavioral deficits such as impaired flight capacity and structural degradation of IFM, the null alleles *up²* and *up³* are particularly severe because they disrupt RNA splicing and eliminate most or all TnT expression in flight and jump muscles (Fyrberg et al., 1990; Viswanathan et al., 2014). Loss of TnT in IFM leads to a complete disruption of myofibrillar organization, with only sparse Z-bands and thin filaments remaining detectable by electron microscopy (Fyrberg et al., 1990). In contrast, the *up (*Chemello et al., 2023*)* mutant selectively affects IFM through defective splicing of a TnT exon 10a isoform, producing disorganized sarcomeres and zebra bodies, a pathological hallmark of human nemaline myopathies (Nongthomba et al., 2007).

Loss of troponin I function produces a distinct but equally severe phenotype. The TnI mutation *heldup-a* (*wupA*) *hdp³* is an IFM-specific TnI null in which sarcomere assembly fails and the affected muscles undergo degeneration (Nongthomba et al., 2004). Despite their similar gross phenotypes, *up¹* and *hdp³* exhibit mechanistically distinct effects on the troponin complex and thin-filament assembly. Whereas the *hdp³* mutant leads to a broad reduction in thin-filament components at both the mRNA and protein levels, the *up¹* mutant selectively disrupts the accumulation of specific proteins, including TpnC4, ubiquitinated Act88F, and the larger TnI isoform that is normally expressed at later stages of IFM development (Nongthomba et al., 2007). These findings demonstrate that troponin components TnT and TnI play essential, yet mechanistically distinct, roles in thin filament regulation and IFM development. Because mutations affecting thin-filament regulation underlie several human myopathies, including nemaline myopathy, these *Drosophila* models provide a useful framework for dissecting how perturbations in troponin function disrupt sarcomere assembly and contractile regulation. Although troponin mutations represent a relatively uncommon cause of nemaline myopathy, the disease more broadly arises from mutations affecting multiple thin-filament proteins. Future studies focusing on troponin mutations, particularly defined point and missense variants, will therefore be important for understanding how distinct molecular perturbations within the thin filament produce overlapping pathological phenotypes.

In addition to skeletal muscle disease, *Drosophila* models have also provided insight into cardiac dysfunction associated with TnT mutations. For example, the *up^101^* mutation disrupts thin-filament regulation and promotes excessive actomyosin activity in the *Drosophila* heart (Fyrberg et al., 1990; Viswanathan et al., 2014). These defects arise from altered myofilament responsiveness to Ca^2+^, a mechanism also implicated in human cardiomyopathies. Similar perturbations in myofilament Ca^2+^ regulation occur in human familial hypertrophic cardiomyopathy caused by mutations in cardiac troponin T (TNNT2), including variants affecting residue Arg92 (Watkins et al., 1995).

Cardiomyocytes also exhibit stretch-sensitive regulation of contraction, and the conserved sarcomeric organization and regulatory mechanisms of *Drosophila* IFM have made it a valuable model for studying disease-causing mutations. A *Drosophila TnT* mutant, analogous to a mutation in cardiac TnT critical for Tm binding, has heart defects that resemble restrictive cardiomyopathy in humans, a result of increased myosin cross-bridges (Wang et al., 2011). EM and 3-D reconstruction of IFM myofilaments indicate that the mutation causes more Tm to be bound to actin, even in the presence of high intracellular Ca^2 + 184^. This study suggests a mechanism of diastolic dysfunction in which the cardiomyocyte is unable to sufficiently relax when TnT is unable to move Tm away from the thin filament (Viswanathan et al., 2014). These findings highlight how impaired regulation of tropomyosin movement by troponin can compromise muscle relaxation. Consistent with this mechanism, pathogenic mutations in tropomyosin directly perturb thin-filament regulation and give rise to a distinct class of myopathies.

### 6.5 Tropomyosin myopathies

The two major tropomyosin genes in *Drosophila*, *Tm1* and *Tm2*, (orthologous to human *TPM1/TPM3* and *TPM2* respectively) are essential for myofibrillar assembly, contractile tuning, and muscle maintenance. Pathogenic variants in *Tm2* (*TPM2*) are established drivers of congenital skeletal muscle disorders, including nemaline myopathy and cap myopathy (Tajsharghi et al., 2007; Mokbel et al., 2013; Citirak et al., 2014). Although more than 30 pathogenic *TPM2* variants have been identified in individuals with myopathies, comprehensive biomechanical characterization has been undertaken for only a small subset. Nearly half of the reported variants remain classified as having uncertain clinical significance (Marttila et al., 2014; McAdow et al., 2022).

Only a limited number of pathogenic *TPM2* variants have been modeled in *Drosophila*, despite their clinical significance in human skeletal muscle myopathies. TPM2 K49Del and E122K are causative mutations associated with congenital myopathies in humans and have been modeled in human cell cultures, where they produce distinct calcium-handling phenotypes (Ohlsson et al., 2008; Tajsharghi et al., 2012; Abdul-Hussein et al., 2013; McAdow et al., 2022). Importantly, *TPM2* mutations associated with similar clinical presentations can exert markedly different effects on thin-filament regulation. For example, the E41K variant reduces myofilament Ca^2+^ sensitivity (Marttila et al., 2014; Avrova et al., 2018) whereas K49Del increases Ca^2+^ sensitivity by destabilizing the relaxed ‘off’ state and promoting hyperactivation (Mokbel et al., 2013). In contrast, a third mutation, E122K, appears to impair productive tropomyosin movement and reduce effective actomyosin interactions (Avrova et al., 2018). Both Tm2 K49Del and E122K have been studied in *Drosophila* larvae and reduce contractile strength and locomotor activity (McAdow et al., 2022). Despite their association with distinct clinical phenotypes in humans, these mutations produce broadly comparable functional and structural defects *in vivo*, indicating that phenotypic severity does not map directly onto clinical classification.

Additional studies in *Drosophila* discovered that a missense mutation in *Tm2* also suppressed TnI hypercontraction and destructive *held-up* phenotypes. An amino acid substitution disrupting TnT-Tm binding (D53 *Tm2; Tm2^S185F^*), homologous to two human cardiac α-Tm mutations implicated in hypertrophic cardiomyopathy, rescues hypercontraction and mobility phenotypes during development and in adult IFM and jump muscle (Naimi et al., 2001). Suppression with D53 *Tm2* partially restores flight and alters Tn-Tm binding and Ca^2+^ sensitivity without fully restoring wild type function (Naimi et al., 2001). Flight defects are only observed in older flies, highlighting the similarity to mutations that cause progressive cardiomyopathies. The D53 *Tm2* substitution likely alters Tn-Tm orientation at the Ca^2+^-sensitive binding interface through introduction of a bulky hydrophobic residue (Naimi et al., 2001). In human hypertrophic cardiomyopathy, a similar mutation in cardiac α-Tm may affect Ca^2+^ sensitivity during tension and force, highlighting the use of *Drosophila* IFM as a useful genetic model in which to study cardiac disease pathogenesis (Naimi et al., 2001).

TPM3 mutations also cause nemaline myopathy and congenital fiber type disproportion (Durling et al., 2002; Ohlsson et al., 2009; Lawlor et al., 2010). Mutations in IFM(3)3 in *Drosophila Tm1* gene (human *TPM3* counterpart) disrupted myofibrils and significantly reduced power output and flight ability (Tansey et al., 1991). Additional studies are needed to systematically model uncharacterized human variants in *Drosophila*, combining contractility, calcium sensitivity, and locomotor assays to identify variant specific pathogenic mechanisms and potential suppressors or therapeutic targets.

Studies of TPM3 and other thin-filament mutations in *Drosophila* underscore how perturbations in myofilament regulation can impair contractile performance without necessarily compromising overall muscle architecture. These models have proven especially valuable for dissecting variant-specific effects on force generation, Ca^2+^ sensitivity, and sarcomere organization. In contrast, muscular dystrophies primarily arise from defects in the structural linkage between the contractile apparatus and the surrounding extracellular matrix, leading to progressive muscle degeneration. This distinction highlights a shift from disorders of contractile regulation to those of mechanical stability and force transmission, which are addressed in the following section.

## 7 Muscular dystrophies

Muscular dystrophies comprise a group of inherited disorders characterized by progressive muscle degeneration, frequently arising from defects in the dystrophin-glycoprotein complex (DGC), which mechanically couples the intracellular contractile apparatus to the extracellular matrix and stabilizes muscle fibers during repeated contraction (Dekkers et al., 2004). In mammals, functional analysis of DGC components is often complicated by genetic redundancy (Dekkers et al., 2004). In contrast, the *Drosophila* genome encodes single orthologs for most DGC proteins, simplifying mechanistic dissection of force-transmission and membrane-stability pathways (Dekkers et al., 2004). The dystrophin gene is highly conserved between vertebrates and invertebrates, enabling functional modeling of dystrophin-associated disease mechanisms in flies (Wasala et al., 2020).

### 7.1 Duchenne and Becker muscular dystrophy

Duchenne muscular dystrophy (DMD) and Becker muscular dystrophy (BMD) are caused by mutations in the dystrophin gene, differing primarily in severity and disease progression (Wasala et al., 2020). *Drosophila* models carrying dystrophin mutations recapitulate key features of DMD pathology, including progressive muscle degeneration, impaired force transmission, and cardiac dysfunction. In flies, dystrophin mutant combinations such as *Dys*^kx43^/*Dys*^Exel16184^ and *Dys*^kx43^/*Dys* produce cardiomyopathy-like defects, providing an *in vivo* system for investigating how defects in cytoskeletal anchoring and membrane stabilization impair cardiac muscle mechanics (Wasala et al., 2020).

These models have also enabled identification of genetic and pharmacological modifiers of disease. Expression of Dp116, a truncated dystrophin isoform, partially rescues cardiac dysfunction in dystrophic flies (Cervia et al., 2024). In addition, administration of plumbagin, a plant-derived vitamin K3 analog, improves climbing ability, reduces oxidative stress, and enhances muscle morphology without detectable adverse effects (Cervia et al., 2024). Mechanistically, plumbagin activates the Nrf2 pathway, restores redox homeostasis, and reduces inflammation, thereby reducing stress-induced muscle damage and improving maintenance of contractile tissue. These findings highlight the utility of *Drosophila* dystrophin models for identifying therapeutic strategies that stabilize muscle integrity and improve resistance to contraction-induced damage (Wasala et al., 2020; Cervia et al., 2024).

### 7.2 Limb-girdle muscular dystrophy

Limb-girdle muscular dystrophy (LGMD) encompasses a heterogeneous group of disorders affecting proximal musculature and is caused by mutations in genes including *LMNA* and *TRIM32*, which encode proteins involved in nuclear integrity and protein turnover, respectively (Bawa et al., 2021; Mohar et al., 2024). Disease-causing mutations in the NHL domain of TRIM32 are structurally and functionally conserved between humans and flies (Bawa et al., 2021; Mohar et al., 2024). Transgenic expression of myopathic *TRIM32* alleles (R394H, D487N, and T520fs) in *Drosophila* induces myofibrillar disorganization, altered nuclear morphology, and reduced TRIM32 protein levels (Bawa et al., 2021).

In a *Drosophila* LGMD2H model expressing patient-derived TRIM32 mutations, muscle pathology is accompanied by overexpression of costamere components, including βPS-integrin and δ-sarcoglycan (Bawa et al., 2021). Similar stoichiometric imbalances are observed in murine myoblasts expressing catalytically inactive TRIM32, suggesting that disruption of integrin-dystrophin-sarcoglycan networks compromises mechanical coupling between the sarcomere, costamere, and extracellular matrix, thereby increasing susceptibility to contraction-induced muscle damage (Bawa et al., 2021). These studies underscore how *Drosophila* models can uncover conserved mechanisms linking protein turnover, cytoskeletal anchoring, mechanotransduction, and maintenance of muscle integrity in muscular dystrophies.

### 7.3 Myotonic dystrophy and splicing dysregulation

In contrast to congenital myopathies, which typically arise from primary mutations in sarcomeric structural or regulatory proteins, myotonic dystrophy reflects a failure of post-transcriptional regulation that secondarily alters myofilament composition, contractile regulation, and muscle function (Lopez-Martinez et al., 2020; Zhang et al., 2024). In myotonic dystrophy type 1 (DM1) and type 2 (DM2), expanded CTG or CCTG repeats in the untranslated regions of *DMPK* and *CNBP* transcripts sequester Muscleblind-like (MBNL) proteins, leading to widespread missplicing of developmentally regulated muscle genes (Warf and Berglund, 2007). A central insight into this mechanism originated from *Drosophila*, where Muscleblind (*mbl*) was first identified as a regulator of muscle development and differentiation (Begemann et al., 1997).

Loss-of-function mutations in *mbl*, including the null allele *mbl*^E27^ and hypomorphic alleles such as *mbl*^ΔE3^, result in severe locomotor defects, disorganized myofibrils, and compromised muscle attachment in somatic and flight muscles (Lopez-Martinez et al., 2020). These phenotypes reflect a failure to properly execute muscle-specific splicing programs rather than primary defects in sarcomere assembly or membrane stability. In addition to genetic loss-of-function, expression of expanded CUG repeat transcripts in *Drosophila* muscle sequesters Mbl protein and phenocopies key features of *mbl* mutants, including impaired locomotion and progressive muscle dysfunction, thereby modeling RNA toxicity observed in human disease (Houseley et al., 2005).

Importantly, *Drosophila* Mbl directly regulates alternative splicing of sarcomeric genes, including *upheld* (*up*, troponin T), where Mbl-dependent inclusion of exon 10A is required for indirect flight muscle integrity (Vicente-Crespo et al., 2008). Disruption of this splicing event in *mbl* mutants leads to IFM-specific defects in sarcomere organization that overlap with phenotypes observed in the TnT splicing mutant *up (1* 106*)*. These findings establish a mechanistic link between Mbl sequestration, missplicing of thin-filament regulatory proteins, impaired sarcomere organization, and muscle dysfunction.

Beyond splicing control, *mbl* mutations also affect muscle maintenance pathways. Overexpression of *mbl* in muscle induces spatially restricted apoptosis through interactions with apoptotic regulators, whereas loss of *mbl* alters polyamine metabolism by disrupting translational control of ornithine decarboxylase (*Odc*) (Marzullo et al., 2023). These additional roles suggest that Mbl integrates splicing regulation with metabolic and survival pathways essential for long-term muscle integrity. Collectively, *Drosophila* models of Mbl dysfunction provide a genetically tractable system for dissecting how chronic splicing dysregulation leads to myotonia and muscle weakness in the despite relatively preserved sarcomere ultrastructure.

## 8 Overarching biomedical significance

Collectively, these disease-focused studies illustrate how disruptions in splicing, metabolism, and sarcomere regulation compromise muscle performance and long-term integrity. Importantly, many of the mechanical and regulatory features uncovered in invertebrate models are not unique to pathological states, but instead reflect conserved principles governing force production, length-dependent activation, and adaptive responses in healthy muscle. Related mechanosensitive regulatory principles are particularly evident in length-dependent activation of cardiac muscle, where conserved sarcomeric processes link passive stretch to increased contractile force. As such, insights gained from insect muscle systems provide a valuable comparative framework for understanding fundamental mechanisms of muscle physiology with broad biomedical relevance.

### 8.1 Length-dependent activation and the Frank-Starling mechanism

Vertebrate cardiac muscle progressively increases contractile force at larger end diastolic volumes, a mechanism that is dependent on sarcomere length and overlap between thick and thin filaments (Kawai and Jin, 2021). Known as the Frank-Starling mechanism, or length dependent activation, sarcomere length-dependent increases in active force are hypothesized to occur via several molecular pathways. Lattice spacing, Ca^2+^ sensitivity, elastic proteins, stiffness, and resting tension may all play a role (Millman, 1998; Fukuda et al., 2008; Farman et al., 2011; Kawai and Jin, 2021). The Frank-Starling response is a cardiac-specific manifestation of length-dependent activation in which changes in sarcomere length alter myofilament Ca^2+^ sensitivity and force production through coordinated structural and mechanical mechanisms (Kawai and Jin, 2021). These observations highlight an important principle shared across muscle systems: structural tuning of thin-filament and elastic proteins can modulate calcium sensitivity and force dynamics (Cao et al., 2019; Cao et al., 2020).

As previously discussed, stretch activation and passive stiffness are readily studied in the unique filament geometry of insect IFM. Insect IFM is composed of relatively rigid, tightly packed sarcomeres with high SA, enabling them to efficiently generate high levels of force. Although the high stiffness of IFM separates it from synchronous vertebrate muscles, the genetic tractability of species like *Drosophila* makes them a useful model to differentiate molecular and mechanical perturbations that increase contractile efficiency and force generation. As such, important discoveries related to dilated cardiomyopathy, restrictive cardiomyopathy and even hypertrophic cardiomyopathy have all come from the study of *Drosophila* IFM (Ayme-Southgate et al., 2005; Viswanathan et al., 2015; Achal et al., 2016). For example, a mutation in α-cardiac actin that causes hypertrophic cardiomyopathy was found to reduce actin flexibility and yield hypercontraction and excessive force in *Drosophila* IFM (Viswanathan et al., 2020). Similarly, a myosin point mutant associated with restrictive cardiomyopathy reduced IFM myofibril stability and implicated myosin head stability and increased myosin ATPase activity and myofilament sliding as potential mechanisms of pathogenesis (Achal et al., 2016). These studies underscore the utility of insect IFM to investigate the common molecular mechanisms underlying passive tension and stretch as they relate to cardiac physiology.

Asynchronous IFM exhibits pronounced stretch-activated behavior, while vertebrate cardiac muscle similarly displays stretch-sensitive regulation of force production. Several lines of evidence suggest that related mechanosensitive processes contribute to the Frank-Starling response, and the molecular mechanisms at play are highly conserved. It will be interesting to further develop tools that are unique to invertebrate flight muscles in order to better understand how sarcomere compliance and passive stiffness contribute to length-dependent activation and efficient force production in vertebrate hearts.

### 8.2 Residual force enhancement

Mechanisms of enhanced force production in lengthening muscle contractions are less well understood than those underlying concentric, or shortening contractions (Herzog, 2014). Eccentric, or lengthening muscle contractions, are characterized by residual force enhancement, where greater force production occurs following active muscle lengthening (Herzog, 2014). Force enhancement occurs at low energy cost and increases with increasing length (up to a maximum amount of stretch) but is reduced when preceded by active shortening (Hisey et al., 2009; Joumaa and Herzog, 2013). Proposed theories of force enhancement are varied and include mechanisms related to cross-bridge cycling, sarcomere length, and passive stiffness of the myofibril (Herzog, 2014). Of particular interest here, evidence suggests that titin and other elastic proteins have an important contribution to force enhancement observed in cardiac muscle.

As discussed previously, the *Drosophila* titin orthologue *sallimus* encodes several modular elastic proteins like kettin, and a separate gene encodes the thick filament-associated protein projectin (Bullard et al., 1985; Bullard et al., 2005). Projectin has orthologues in other invertebrate species like *C. elegans* and the mollusc *Mytilus*, supporting a conserved role in muscle contraction, resting elasticity and extensibility (Bullard et al., 2005). One residual force enhancement hypothesis bolstered by invertebrate studies is the winding filament theory. This hypothesis explains enhanced force during eccentric contraction at low energy cost, theorizing that active stretch extends elastic proteins, storing elastic energy without the need for ATP (Joumaa and Herzog, 2013). In this model, active stretch elongates the PEVK region of elastic proteins, allowing storage of elastic energy during eccentric contraction, while interactions between cross-bridges and thin filaments contribute to force enhancement in shortened muscle (Monroy et al., 2012).

Insect muscles harbor enormous structural and functional diversity between sarcomeres, but the relationship between the length of giant elastic proteins and their stiffness appears highly conserved. The stiff indirect flight muscles express smaller isoforms of elastic proteins, similar to cardiac titin in vertebrates (Lindstedt and Nishikawa, 2017). Complex genetic studies in *Drosophila* IFM make it possible to link molecular and structural components of the sarcomere to physiological processes like residual force enhancement *in vivo.* Studies in the IFM from insects such as *Drosophila* and *Lethocerus* provide an opportunity for precise dissection of the biochemical and biomechanical mechanisms driving processes like residual force enhancement at the organismal level, adding to our understanding of variable stiffness and muscle activation in vertebrate hearts. Together, these principles of length-dependent activation and force enhancement define how muscles adapt acutely to mechanical demands. Over time, however, the capacity to sustain and regulate these responses becomes a central determinant of muscle aging and longevity.

### 8.3 Aging and longevity

Aging is accompanied by a progressive decline in muscle performance that precedes overt tissue degeneration and reflects cumulative defects in contractile regulation, mechanical resilience, and cellular maintenance. In both skeletal (Demontis and Perrimon, 2010; Demontis et al., 2013) and cardiac muscle (Wessells et al., 2004; Ocorr et al., 2007a; Ocorr et al., 2007b; Wessells and Bodmer, 2007a; Ocorr et al., 2014), age-associated dysfunction emerges from impaired sarcomere stability (Mery et al., 2008), altered Ca^2+^ handling (Viswanathan et al., 2014), and reduced capacity to adapt to mechanical stress (Taghli-Lamallem et al., 2014; Chechenova et al., 2024). *Drosophila* provides a useful system in which to dissect these processes, as age-dependent changes in muscle structure and function can be measured longitudinally and genetically uncoupled from developmental defects.

Age-related cardiac dysfunction has been particularly well characterized in *Drosophila* and reveals striking parallels with mammalian cardiac aging (Wessells and Bodmer, 2004; Wessells et al., 2004; Sujkowski and Wessells, 2015; Cannon et al., 2017; Blice-Baum et al., 2019). Early studies demonstrated that aging flies exhibit progressive declines in cardiac performance, including increased arrhythmias, impaired stress tolerance, and heightened susceptibility to pacing-induced failure (Wessells et al., 2004), establishing *Drosophila* as a powerful model for studying cardiac aging *in vivo (*Wessells and Bodmer, 2004; Wessells and Bodmer, 2007b). Importantly, these findings showed that age-dependent cardiac dysfunction reflects deterioration of contractile regulation and mechanical performance rather than loss of cardiomyocytes, providing a mechanistic framework for understanding how muscle aging emerges from failures in maintenance rather than development (Luong et al., 2006; Ocorr et al., 2007b; Wessells et al., 2009; Lim et al., 2011).

Skeletal muscle aging in *Drosophila* similarly involves progressive functional decline accompanied by disruptions in sarcomere organization and myofibrillar maintenance (Gargano et al., 2005; Demontis et al., 2013; Chechenova et al., 2024). Age-dependent reductions in flight and climbing ability correlate with Z-disc disorganization (Mery et al., 2008), altered filament spacing (Achal et al., 2016; Rastegarpouyani et al., 2024), and compromised proteostasis (Demontis and Perrimon, 2010; Hunt et al., 2025) in the indirect flight muscle. Importantly, these changes are not simply the result of reduced expression of contractile proteins, but instead reflect impaired turnover (Demontis and Perrimon, 2010; Bai et al., 2013), mitochondrial dysfunction (Owusu-Ansah et al., 2013; Lee et al., 2021), and declining capacity to buffer mechanical and metabolic stress (Lee et al., 2010; Bai et al., 2013). These features mirror key aspects of age-related muscle weakness and sarcopenia in vertebrates and highlight the importance of maintenance pathways in preserving muscle function across the lifespan.

Mitochondrial dysfunction and redox imbalance are central features of skeletal muscle aging across species, including *Drosophila*. In flies, age-related declines in mitochondrial respiration are accompanied by altered expression of genes involved in oxidative phosphorylation and the tricarboxylic acid cycle, as well as structural abnormalities such as mitochondrial enlargement and cristae disorganization (Ferguson et al., 2005; Girardot et al., 2006). Variation in mitochondrial DNA haplotypes influences mitochondrial bioenergetic capacity, reactive oxygen species production, mitochondrial DNA copy number, and organismal lifespan, with aging further exacerbating these effects (Correa et al., 2012; Camus et al., 2015; Oka et al., 2015; Del Campo et al., 2016). Consistent with a causal role for mitochondrial function in muscle aging, muscle specific genetic modulation of the mitochondrial aspartate transporter Ucp4a extends lifespan by preserving muscle health and function (Baek et al., 2025).

As mitochondrial dysfunction and oxidative stress accumulate with age, muscle fibers become increasingly susceptible to damage and degeneration through multiple cellular pathways. Accumulation of oxidative damage with advanced age increases apoptosis in flight and leg muscles (Zheng et al., 2005). However, more recent studies demonstrate that adult muscle degeneration can proceed through non-apoptotic mechanisms, including sarcolemmal rupture, mitochondrial swelling, and segmental damage consistent with necrotic cell death (Chechenova et al., 2024). These findings raise important questions about how age-associated oxidative stress and mitochondrial dysfunction predispose muscle fibers toward distinct modes of cell death (Zheng et al., 2005; Chechenova et al., 2024). Future studies will be needed to identify the genetic and metabolic checkpoints governing this switch and to determine whether stabilizing membrane integrity or restoring redox homeostasis can prevent necrotic degeneration and preserve muscle function during aging.

Reduced physical activity is another key contributor to age-related muscle decline (Zhang et al., 2007; Brener et al., 2023). A *Drosophila* model of long-term inactivity demonstrated that limitation of movement shortens lifespan, reduces climbing and endurance performance, and induces structural defects in actin filaments within the indirect flight muscle (Protasiewicz et al., 2025). This model provides a useful platform for studying disuse-induced muscle atrophy and its interaction with aging-related muscle degeneration (Protasiewicz et al., 2025).

Exercise, conversely, provides an experimental lens through which to examine the plasticity of aging muscle and heart. Endurance training paradigms in *Drosophila* improve locomotor performance, enhance stress resistance, and preserve both skeletal and cardiac muscle function with age (Piazza et al., 2009; Sujkowski and Wessells, 2018). Exercise induces conserved metabolic and transcriptional adaptations (Sujkowski et al., 2015; Mendez et al., 2016), including improved mitochondrial quality control (Laker et al., 2014), enhanced redox balance (Kim et al., 2020), and stabilization of sarcomere architecture (Protasiewicz et al., 2025). Notably, exercise benefits extend to aged animals (Piazza et al., 2009) and disease models (Sujkowski et al., 2012; Sujkowski et al., 2022), demonstrating that age-associated muscle decline is modifiable rather than inevitable. These findings underscore the value of invertebrate systems for identifying conserved pathways through which physical activity promotes muscle resilience and longevity.

Collectively, studies of muscle aging in *Drosophila* reinforce the idea that long-term muscle health depends on the coordinated regulation of contractile mechanics, elastic properties, and cellular maintenance pathways. Aging amplifies vulnerabilities in thin-filament regulation, elastic scaffolding, and Ca^2+^-dependent activation, providing a unifying context in which regulatory, structural, and metabolic processes intersect. By enabling precise genetic and physiological dissection of these interactions over time, invertebrate models offer unique insight into the mechanisms that govern muscle aging and identify potential strategies for preserving muscle function across the lifespan.

## 9 Mechanistic gaps and future directions

Insects have evolved highly specialized muscle systems that reveal fundamental principles of myofilament regulation, mechanical tuning, and energy-efficient force production. Stretch activation in asynchronous flight muscle represents one extreme solution, in which contraction is sustained at low, priming levels of Ca^2+^ and mechanical feedback rather than neural input drives oscillatory work. By cycling each wing beat out of phase with motor neuron firing, insects minimize the energetic costs associated with Ca^2+^ handling, ATP turnover, and repeated activation and relaxation. However, stretch activation should be viewed not as an isolated specialization, but as part of a broader continuum of length-dependent and mechanically regulated behaviors that are conserved across striated muscles.

Insights into these processes have been shaped by studies in invertebrate systems such as *Lethocerus*, *Bombus*, and *Drosophila*, which offer complementary experimental advantages. Comparative analyses of thin-filament regulation, elastic protein function, and sarcomere architecture in these models have revealed how passive tension, filament compliance, and regulatory positioning are tuned to meet distinct mechanical demands. At the same time, incomplete evolutionary conservation highlights key structure-function relationships that constrain isoform interchangeability across species and muscle types. Understanding why certain invertebrate specializations cannot substitute for vertebrate counterparts will be critical for defining the molecular logic that links passive mechanical properties to myofilament regulation.

Across systems, these studies underscore that passive stretch, elastic recoil, and length-dependent activation are integral components of muscle function in both health and disease. By leveraging the genetic and mechanical tractability of insect muscle, future work will continue to refine our understanding of how myofilament regulation, passive tension, and sarcomere stability interact across physiological contexts and contribute to heritable myopathies and age-related muscle dysfunction.

## Glossary

ACTA1: α-skeletal actin 1
ACTC1: α-cardiac actin 1
ATP: adenosine triphosphate
BMD: Becker muscular dystrophy
Ca^2+^: calcium ion
CM: congenital myopathy
CNBP: cellular nucleic acid binding protein
DA1: distal arthrogryposis type 1
DA2B: distal arthrogryposis type 2B
DGC: dystrophin–glycoprotein complex
DLM: dorsal longitudinal muscle
DM1: myotonic dystrophy type 1
DM2: myotonic dystrophy type 2
DMD: Duchenne muscular dystrophy
DVM: dorsal ventral muscle
EF-hand: helix-loop-helix calcium-binding motif
Fn: fibronectin domain
HCM: hypertrophic cardiomyopathy
IFM: indirect flight muscle
Ig: immunoglobulin domain
IBM-3: inclusion body myopathy type 3
LDA: length-dependent activation
LGMD: limb-girdle muscular dystrophy
MBNL: Muscleblind-like protein
Mbl: Muscleblind
Mhc: myosin heavy chain gene (*Drosophila*)
Odc: ornithine decarboxylase
PEVK: proline–glutamate–valine–lysine domain
SA: stretch activation
SD: shortening deactivation
Sls: sallimus (*Drosophila* titin homolog)
SR: sarcoplasmic reticulum
rFE: residual force enhancement
TDT: tergal depressor of the trochanter
Tm: tropomyosin
Tn: troponin
TnC: troponin C
TnH: heavy troponin
TnI: troponin I
TNNT2: vertebrate cardiac troponin T
TnT: troponin T
